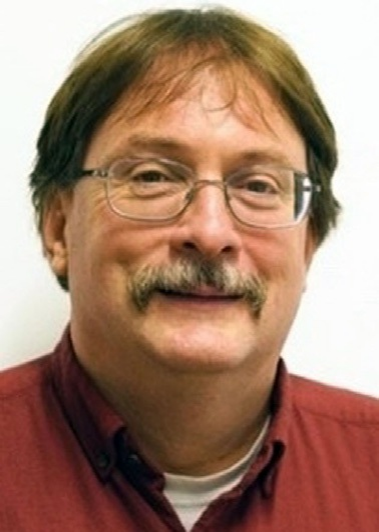# Mononuclear Dysprosium Alkoxide and Aryloxide Single‐Molecule Magnets

**DOI:** 10.1002/chem.202100085

**Published:** 2021-03-24

**Authors:** Vijay S. Parmar, David P. Mills, Richard E. P. Winpenny

**Affiliations:** ^1^ Department of Chemistry The University of Manchester Oxford Road Manchester M13 9PL UK

**Keywords:** alkoxides, aryloxides, lanthanides, magnetic properties, single-molecule magnets

## Abstract

Recent studies have shown that mononuclear lanthanide (Ln) complexes can be high‐performing single‐molecule magnets (SMMs). Recently, there has been an influx of mononuclear Ln alkoxide and aryloxide SMMs, which have provided the necessary geometrical control to improve SMM properties and to allow the intricate relaxation dynamics of Ln SMMs to be studied in detail. Here non‐aqueous Ln alkoxide and aryloxide chemistry applied to the synthesis of low‐coordinate mononuclear Ln SMMs are reviewed. The focus is on mononuclear Dy^III^ alkoxide and aryloxide SMMs with coordination numbers up to eight, covering synthesis, solid‐state structures and magnetic attributes. Brief overviews are also provided of mononuclear Tb^III^, Ho^III^, Er^III^ and Yb^III^ alkoxide and aryloxide SMMs.

## Graphical abstract

**Mononuclear lanthanide single‐molecule magnets** (SMMs) have progressively evolved in recent years. Lanthanide alkoxide and aryloxide chemistry offers a modular design approach to study the relaxation dynamics of high‐performing SMMs. Alkoxide and aryloxide ligands on a single axis at a Dy^III^ ion enhances the magnetic anisotropy and the barrier to magnetic reversal (*U*_eff_). Leading examples of Dy^III^ alkoxide and aryloxide SMMs are discussed in this review.

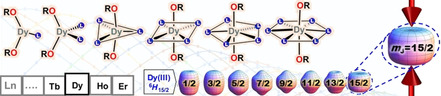

## 1 Introduction

**Figure 1:**
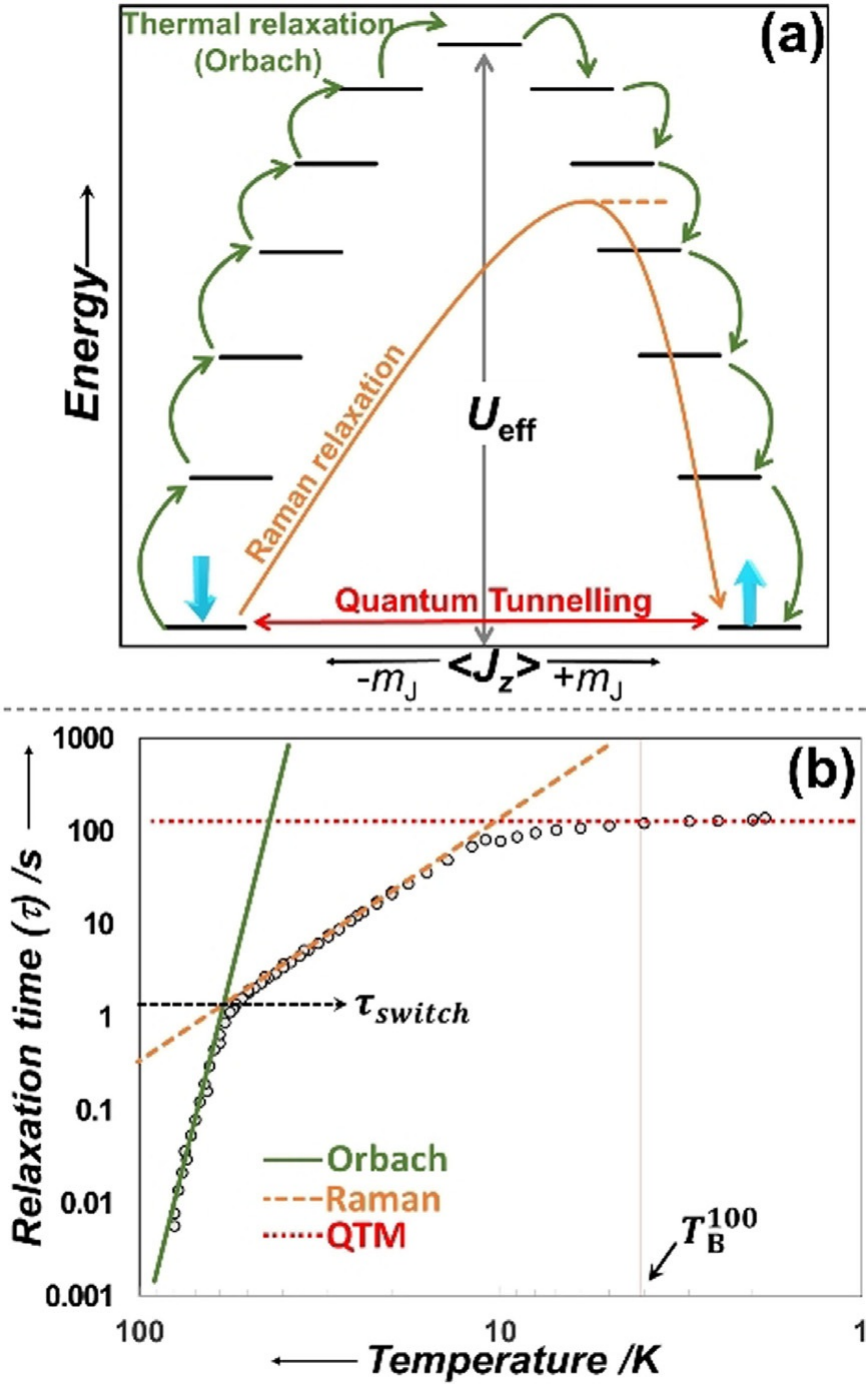
(a) Schematic of the energy barrier (*U*_eff_) and the main relaxation mechanisms. The black lines represent the energies of various magnetic spin states (*m*_J_). The spin trapped in one of the bistable *m*_J_ states relaxes back (crossing the barrier: *U*_eff_) to its original state by either stepwise transitions through higher energy *m*_J_ states (i.e., Orbach process; green arrows), by going through a virtual excited state (Raman; orange arrow) or by quantum tunnelling of magnetisation (QTM; red arrow) from the ground state. (b) Relaxation profile (τ vs. *T* ^−1^) of an SMM showing the different temperature regions where one relaxation process dominates over the others. Orbach (green line) process at higher temperatures, Raman (orange dashed line) in the intermediate range and QTM (red dotted line) in the low temperature region. The black circles represent the observed/measured τ value against temperature. τ_switch_ is the relaxation time at which the Raman process and the Orbach process have the same relaxation rate. $T_{B}^{100}$ is the temperature at which τ values are over 100 s.

The discovery in 1993^[1]^ that a Mn_12_ cluster could retain magnetisation for significant time periods have inspired numerous collaborative efforts between chemists and physicists to develop a new class of materials.[^2^, ^3^] Molecules that show slow relaxation of magnetisation of purely molecular origin are called single‐molecule magnets (SMMs),^[2]^ and their unique magnetic properties could be applied to the development of quantum computers, molecular spintronics and ultra‐high‐density data storage devices.^[3]^ SMMs work by trapping the magnetic spins in one of the bistable magnetic states under an external magnetic field. Upon removal of the external field, the trapped spins slowly relax back to their original state (Figure 1; see Section 2 for a brief overview of the magnetic relaxation processes). The barrier that the spin crosses to relax is known as the effective energy barrier to magnetic reversal (*U*_eff_), and the temperature under which this relaxation process remains blocked is the blocking temperature (*T*_B_) of that SMM.^[2]^

For a decade many large d‐transition metal molecular clusters like the first Mn_12_ SMM were synthesised.^[1]^ However, it was shown that such clusters tend to diminish the overall magnetic anisotropy (directional dependence of magnetic susceptibility), which is integral to SMM properties.^[4]^ Following the discovery of a terbium phthalocyanine SMM (**TbPc2**) in 2003, mononuclear lanthanide (Ln) SMMs with enhanced magnetic behaviour were realised.^[5]^ Soon after, the SMM community was directed towards synthesising Ln SMMs containing a single Dy^III^ centre in a uniaxial ligand field environment to achieve the highest performing Ln SMMs.^[6]^

Due to the large radii of Ln^III^ ions and their electrostatic bonding, they are inclined to form high coordination number (CN) complexes, with a lack of geometrical control.^[7]^ Sterically demanding ligands are required to achieve Ln complexes with low CNs by controlling the Ln coordination sphere.^[8]^ In 2011 the Manchester group examined using a sterically demanding alkoxide, and made {Dy_5_},^[9]^ {Ho_5_}^[10]^ and {Dy_4_K_2_}^[11]^ cages where the Ln sites were six‐coordinate; these had high *U*_eff_ values as the highly axial crystal field led to relaxation via the second and third excited states.^[11]^

In 2016 Tong and co‐workers utilised the anionic chelating aryloxide ligand bbpen^2−^ (*N*,*N*′‐bis(2‐methylenepyridinyl)ethylene‐diamide) to synthesise a mononuclear Dy^III^ SMM with a pentagonal bipyramidal (PB) geometry that showed the first energy barrier to magnetisation reversal >1000 K.^[12]^ In the same year, Ding et al. reported another high‐performing PB Dy SMM [Dy(O*t*Bu)_2_(py)_5_][BPh_4_] (py=pyridine), exhibiting a *U*_eff_ of 1800 K, setting a new record.^[13]^ These recent increases in *U*_eff_ for Ln SMMs have facilitated measurements of the finer details of SMM relaxation processes,[^12^, ^13^, ^14^, ^15^, ^16^, ^17^, ^18^] showing that the low energy vibrational modes close to the metal centre can couple with low energy acoustic phonons to promote the Raman relaxation process.^[19]^ More studies of this nature are needed to deepen our understanding of the relaxation dynamics of high‐performing SMMs, and Ln alkoxides and aryloxides have proved to be useful test subjects.

Ln alkoxide and aryloxide chemistry is rich and well‐developed, with hundreds of mono‐ and multi‐nuclear structurally authenticated complexes reported.[^11^, ^20^, ^21^, ^22^, ^23^, ^24^, ^25^, ^26^] Previous reviews have provided extensive discussions of mono‐ and multi‐nuclear Ln SMMs, including non‐alkoxide and aryloxide ligands,[^27^, ^28^, ^29^, ^30^] and general Ln alkoxide and aryloxide chemistry.[^20^, ^31^, ^32^] For the purposes of this review, we focus on mononuclear Dy^III^ alkoxide and aryloxide SMMs, with other mononuclear heavy Ln systems covered briefly. We will firstly provide a brief summary of the relaxation dynamics of SMMs, providing the reasons for mononuclear Ln SMMs delivering the highest *U*_eff_ values to date. We then give an overview of general Ln alkoxide and aryloxide chemistry before covering mononuclear Ln SMMs of these ligands, with the most important Dy examples described in detail.

## 2 Relaxation Dynamics and Magnetic Blocking

Magnetic relaxation occurs when a spin (magnetic moment of the spin) interacts with other spins in the crystal or the medium (phonons); here phonons are quantised vibrations of the crystal lattice, that is energy packets of fixed frequencies that are generated by the movement of the lattice in the crystal. These vibrations cause movement of the atoms and their charges. The varying charges alter the local electromagnetic field (crystal field) acting upon the spin, providing it with the energy needed to reorient itself and relax. This coupling of the electronic spin with the lattice vibrations is known as spin‐phonon coupling, which causes the transition of the spin from one total magnetic spin state (*m*_J_) to another.[^33^, ^34^]

The magnetic relaxation process constitutes multiple individual relaxation steps depending upon the nature and symmetry of the ligand field environment about the magnetic metal centre as well as the choice of the magnetic ion.[^33^, ^35^] Equation 1 governs the relaxation dynamics of SMMs; τ^−1^ is the rate of magnetic relaxation encompassing all the individual relaxation processes at any temperature *T* (Figure 1).[^2^, ^33^] It is to be noted that Equation (1) is an approximation based on the work of Orbach from the 1960s[^36^, ^37^] and alternate versions exist in the literature, as there is a variable functional dependence of the individual relaxation terms. The first term represents the Orbach relaxation process, giving the energy barrier, *U*_eff_; where *K*_B_ is the Boltzmann constant and $\tau_{0}^{-1}$ is the exponential rate constant of the Orbach process.^[36]^ The Orbach relaxation process requires a stepwise thermal relaxation process by going through all the 2*J*+1 (*J*=*L*+*S*, total angular momentum quantum number) individual states for Ln.^[36]^ The Orbach mechanism generally operates at relatively high temperatures.^[33]^

(1) $$\tau^{-1}=\;\tau_{0}^{-1}\exp\left(-\frac{U_{eff}}{K_{B}T}\right)\;+{CT}^{n}+\tau_{QTM}^{-1}\;$$

The second term in Equation (1) represents the Raman process; it is parametrised by the power‐law expression *CT* ^*n*^ where the constant term *C* is a combination of multiple terms including the matrix elements of the crystal field (CF) potential, which is related to the spin‐phonon coupled transition from one magnetic state to another.^[37]^ The power *n* depends upon the distribution of phonon modes in the crystal; it was predicted to be ≈9 for Kramers ions (odd number of valence electrons) such as Dy^III^,[^36^, ^37^] however, it has been found to vary from 2–5 in the majority of SMMs.[^13^, ^14^, ^15^, ^38^] The Raman relaxation mechanism involves the absorption and emission of a high‐energy phonon to reach a non‐stationary virtual state before relaxing to the ground or low‐lying excited state.[^33^, ^37^] This mechanism is generally dominant in the intermediate temperature range.^[33]^

The third term in Equation (1) represents the temperature‐independent quantum tunnelling of magnetisation (QTM) process.[^2^, ^33^, ^39^] QTM generally occurs at lower temperatures in the absence of external magnetic field when the spin does not have sufficient energy to cross the barrier via a thermal energy exchange process. QTM proceeds by resonance between the states at either side of the barrier,[^1^, ^39^] and normally happens at the ground state but can also occur at excited states in combination with a thermal relaxation process; this is known as thermally assisted QTM (TA‐QTM).^[40]^ QTM is usually hindered in Kramers ions; however increased rhombicity can enhance QTM. To improve the efficiency of SMMs, we need to slow all of these relaxation processes. The thermal energy barrier, *U*_eff_, is only directly relevant to one of three processes.

**Figure 2:**
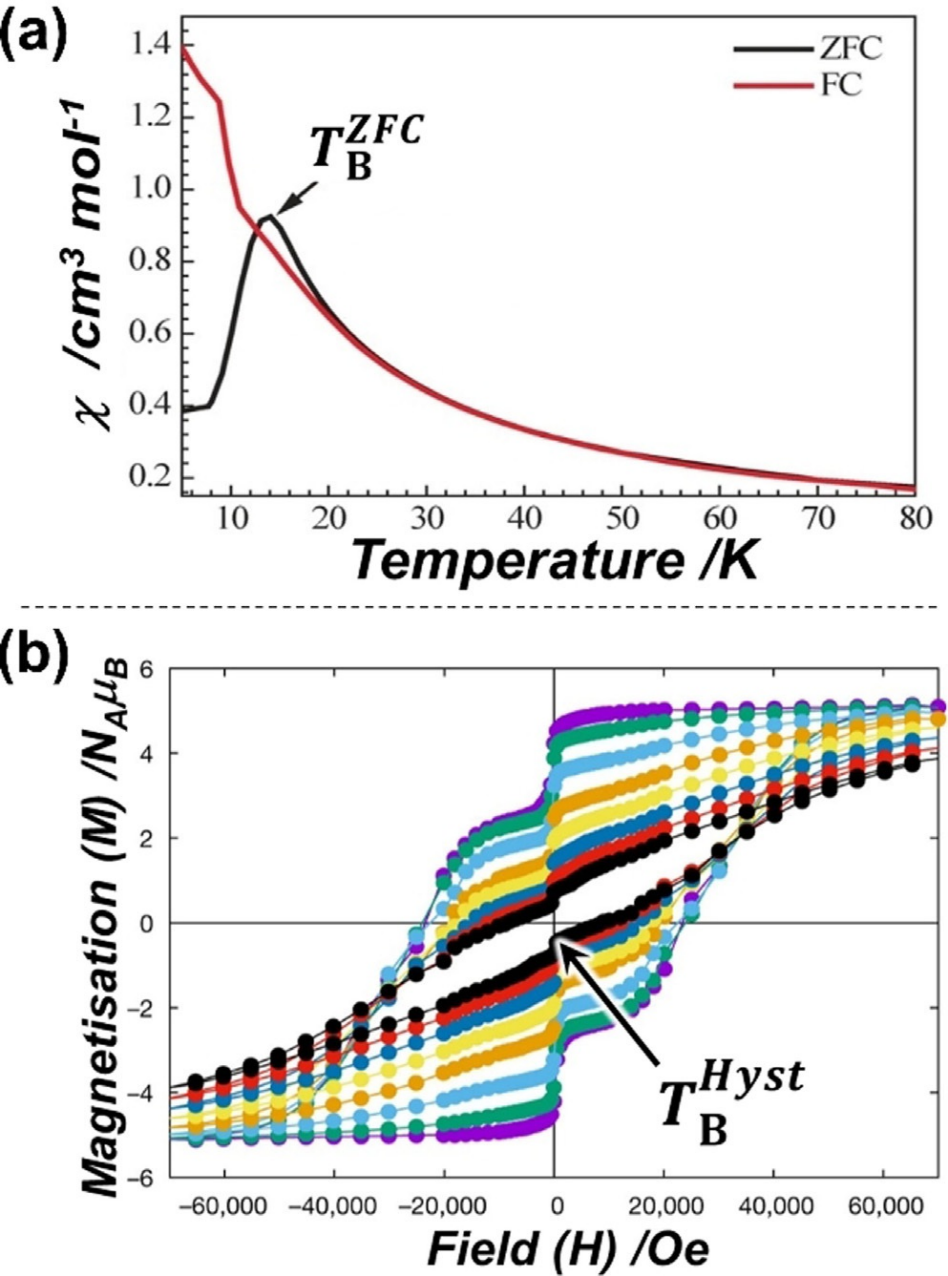
Plots to define the blocking temperatures $T_{B}^{ZFC}$ (a)^[13]^ and $T_{B}^{Hyst}$ (b).^[38]^ (a) A plot of molar magnetic susceptibility (χ) vs. *T*. In the ZFC (zero‐field cooled, black line) mode the sample is first cooled down to 1.8 K in the absence of an external magnetic field, then χ is measured under an external magnetic field with respect to increasing temperature. The FC (field cooled, red line) mode is when χ is measured under an external field while the sample is being cooled down from room temperature to 1.8 K. The temperature at which a peak in the ZFC‐mode χ vs. *T* plot is observed is defined as $T_{B}^{ZFC}$ . (b) Isothermal curves of magnetisation (*M*) vs. external field (*H*) at multiple temperatures (lowest temperature in purple, highest temperature in black, with other colours stepwise differences in temperature between these values); these are also known as magnetic hysteresis loops. The highest temperature at which the hysteresis loop remains open (positive coercive field) is $T_{B}^{Hyst}$ which strongly depends upon the field sweep rate. Adapted with permission from, (a) ref. [13], copyright 2016 Wiley‐VCH, and (b) ref. [38], copyright 2017, Nature Publishing Group.

Experimentally, we characterise SMMs by measuring the magnetic susceptibility against temperature at multiple frequencies in an alternating current (AC) magnetic field; the relaxation times ***τ*** can be extracted from these data. The ***τ*** values plotted against temperature provides the relaxation profile shown in Figure 1 b. At any temperature, the ***τ*** value is a combination of all the individual relaxation processes. This relaxation profile is fitted using Equation (1), and from the high‐temperature region, the *U*_eff_ value (green line) (Figure 1 b) can be extracted. The same relaxation plot also provides the blocking temperature, *T*_B_; this has many definitions in the literature. The textbook definition of *T*_B_ is shown in Figure 2 a, that is, the maximum in the zero‐field magnetic susceptibility.[^2^, ^13^] This definition is traditionally used in superparamagnetic nanoparticles and is denoted $\;T_{B}^{ZFC}$ here. An alternate definition has been proposed, $T_{B}^{100}$ , which is the temperature at which the ***τ*** value reaches 100 s and is shown in Figure 1 b. By measuring the magnetic hysteresis loops (magnetisation, *M*, vs. external field, *H*) at multiple temperatures, the temperature at which the loop remains open is defined as the hysteresis blocking temperature ($T_{B}^{Hyst}$ ) of that SMM (Figure 2 b).[^2^, ^38^]

## 3 Why Mononuclear Lanthanide SMMs?

**Figure 3:**
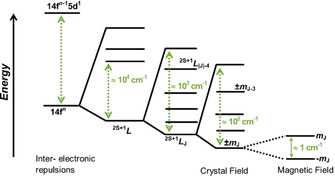
Electronic interactions in Ln valence orbitals and their typical magnitudes, showing effects of weaker perturbations resulting in the splitting of degenerate states.^[28]^ A 1 T magnetic field is assumed here.

Magnetic anisotropy arises from the interaction of electronic spin and angular momentum.^[28]^ The angular momentum of Ln 4f orbitals remains unquenched and can couple with 4f electron spins, thus producing large magnetic anisotropy.[^28^, ^33^] The recent mononuclear SMM approach takes advantage of the physical properties of Lns.[^12^, ^13^, ^38^] The energy of spin‐orbit coupling is greater than the CF in Ln SMMs.^[28]^ Thus, the electronic structure of the Ln ion requires that the spin‐orbit coupled ground state term symbol be used.[^28^, ^33^] The interaction of the spin‐orbit coupled quantum state *J* (*J*=*L*+*S*; where *L* and *S* are the total orbital and spin angular momentum quantum numbers, respectively) with the CF contributes to the rise of magneto‐crystalline anisotropy, which in turn affects the energy barrier (Figure 3).[^28^, ^33^]

Considering Dy^III^ as an ion of especial interest for mononuclear Ln SMMs, the ground state *J* of the free ion has 2*J*+1 degenerate states, *m*_J_=±15/2, ±13/2, ±11/2, ±9/2, ±7/2, ±5/2, ±3/2, ±1/2. These are the projections of total angular momentum on a single axis. The CF from the charges and ligand atoms surrounding the magnetic ion will affect these 2*J*+1 projections, thereby breaking the degeneracy of the ground state (Figure 3).[^6^, ^28^, ^33^] This breaking of the spin‐orbit coupled states via magnetic anisotropy introduced by the ligand field suggests that the magnetic properties of a complex can be modulated through ligand design.

The use of symmetry in low coordinate Ln SMMs to modulate the CF to achieve improved SMM behaviour has been discussed exhaustively in the literature.[^14^, ^17^, ^35^, ^41^] From group theory, a general form of the CF within the *J* multiplets of the 4f ions can be expressed via the Hamiltonian, $H_{CF}=\sum_{k=2,4,6}\sum_{\left(q=-k\right)}^{k}B_{k}^{q}\;O_{k}^{q}\left(\;J\right)$ , where $O_{k}^{q}\left(\;J\right)$ are functions of the total angular momentum *J*, (also known as Stevens operators). $B_{k}^{q}$ represents the CF parameters, k is the order of the Stevens operator, and *q* ranges from −*k* to *k*. $B_{k=0}^{q}$ are the axial parameters and conversely, $B_{k\neq 0}^{q=2,4,6}$ are the transverse parameters. An ideal point group symmetry such as *C*_∞v_, *D*_4*d*_, *S*_8_, *D*_5*h*_, *D*_6*d*_, and *D*_∞h_ could greatly suppress the unwanted QTM process, though these precise geometries are unlikely to be realised in molecular complexes.

**Figure 4:**
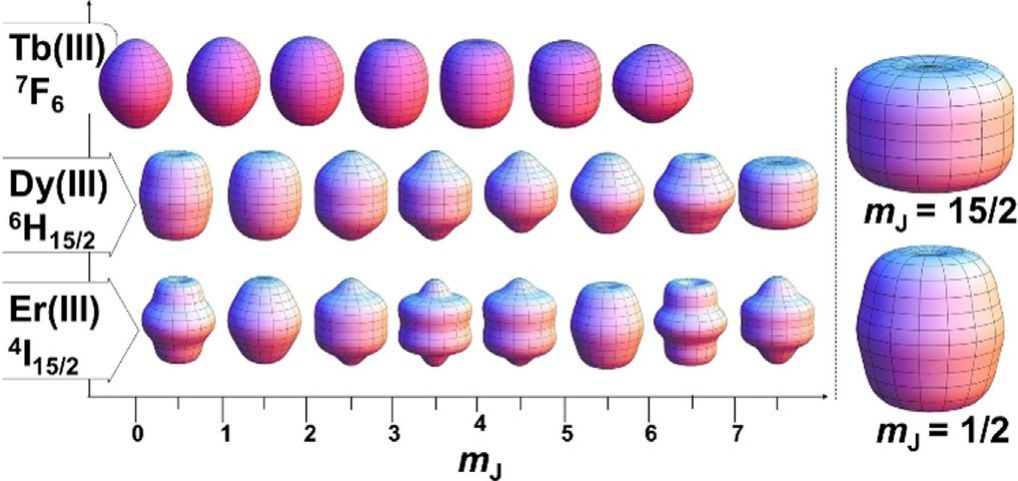
Approximated charge densities of various magnetically degenerate *m*_J_ states of Ln^III^ ions for Tb, Dy and Er; the most (*m*_J_=15/2) and least (*m*_J_=1/2) magnetic states of Dy^III^ are expanded on the right of the diagram.[^6^, ^42^]

The extent of magnetic anisotropy in Ln depends upon the distribution of electron density at the ground *m*_J_ level.^[6]^ In 1981, Sievers published the electron density maps of different magnetic states of Ln^III^ ions (Figure 4 shows the *m*_J_ states of Tb^III^, Dy^III^ and Er^III^).^[42]^ Based on this electron density distribution of the *m*_J_ levels in various Ln^III^ ions, different CFs (axial or equatorial) are required to stabilise the largest *m*_J_ level as the ground state.^[6]^ This divides the Ln^III^ ions into so‐called oblate (Ce^III^, Pr^III^, Nd^III^, Tb^III^, Dy^III^ and Ho^III^) and prolate (Pm^III^, Sm^III^, Er^III^, Tm^III^ and Yb^III^) ions; the oblate ions prefer axial CF, whilst the prolate ions require equatorial CF.^[6]^

For designing high‐performing Ln SMMs, the Ln ion should have a doubly‐degenerate magnetic ground state (for the existence of magnetic bi‐stability), and this ground state should be the maximum of all the *m*_J_ states that the ion possesses. This is to extract the maximum magnetic moment from the ground state, which will be mostly populated at sufficiently low temperatures (Boltzmann distribution). Of all the Lns, Dy^III^ is ideal for Ln SMMs as it is a Kramers ion, which provides a doubly degenerate ground state, and its ^6^H_15/2_ term provides the highest quantum magnetic ground state spin.[^28^, ^33^] For strong anisotropy, the ground *m*_J_ state and the first excited state *m*_|J|‐1_ should be well‐separated. This separation determines how slowly the spin can relax via temperature‐dependent mechanisms in the absence of QTM.[^28^, ^33^] The oblate shape (Figure 4, top right) of the *m*_J_=±15/2 state of the Dy^III^ ion suggests that uniaxial charges applied from the top and bottom of this state would stabilise it so that it becomes the magnetic ground state.^[6]^ The same uniaxial ligand field also destabilises the prolate‐shaped *m*_J_=±1/2 state (Figure 4, bottom right), hence increasing the separation between the ground and first excited state, and therefore the overall energy barrier.^[6]^

**Figure 5:**
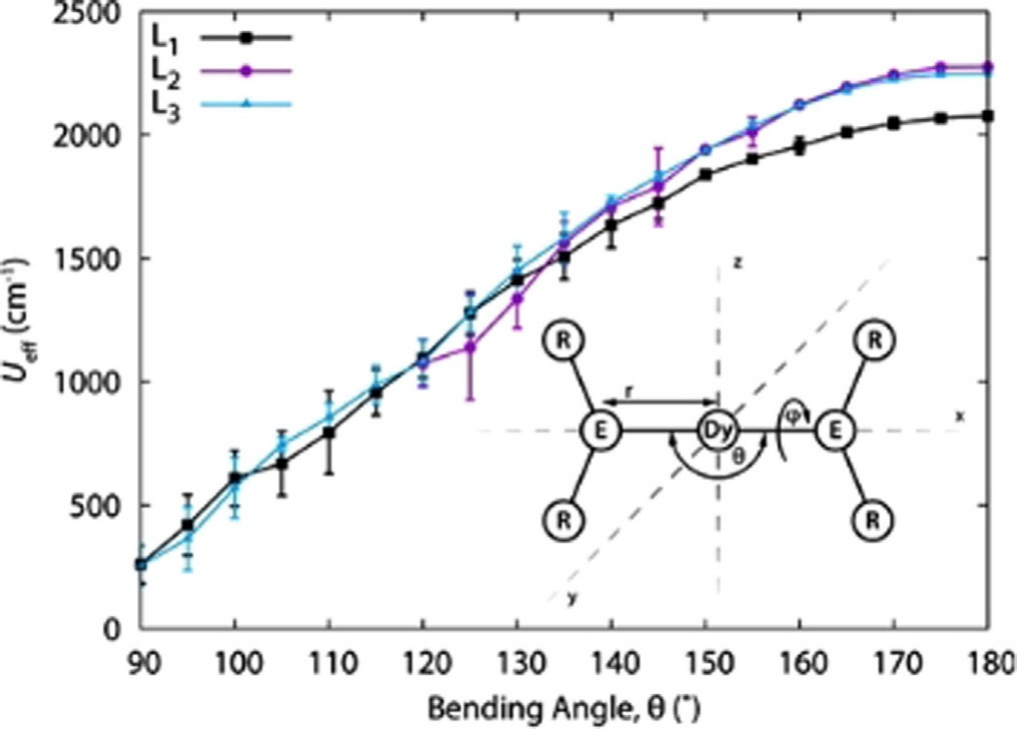
Energy barrier (*U*_eff_) for hypothetical [Dy(L)_2_]^+^ cations {ER_2_=L: L_1_=N(SiH_3_)_2_, L_2_=C(SiH_3_)_3_, L_3_=CH(SiH_3_)_2_} with respect to E‐Dy‐E angle (*θ*, E=N or C). Taken with permission from ref. [54], copyright 2015, American Chemical Society.

In 2015 Chilton et al. reported^[43]^ computational results verifying this hypothesis, using the coordinates of the crystal structure of a near‐linear Sm^II^ complex [Sm{N(Si*i*Pr_3_)_2_}_2_]. An extension of these studies related the E‐Dy‐E (E=donor atom) angle with the magnitude of *U*_eff_ and predicted a barrier higher than 2000 K (Figure 5) for a hypothetical near‐linear Dy^III^ cation [Dy{N(SiH_3_)_2_}_2_]^+^ with no donor atom coordination at the equatorial positions.^[44]^ Similar calculations had been performed by Chibotaru and Ungur,^[45]^ and the Rajaraman group,^[46]^ but on chemically unfeasible materials.

Using [Dy(O*t*Bu)_2_(py)_5_][BPh_4_] as an example, this complex has two strongly coordinating anionic alkoxide ligands (*tert*‐butoxides) situated on a single axis with a O‐Dy‐O angle of 178.91(9)°, with five neutral pyridine molecules coordinated in the equatorial plane.^[13]^ The notably high *U*_eff_ (1815 K) in [Dy(O*t*Bu)_2_(py)_5_][BPh_4_], despite the presence of five equatorial donor atoms, proves that strong donor ligands like alkoxides are favourable for the synthesis of improved mononuclear Dy SMMs.

## 4 Ln Alkoxide and Aryloxide Chemistry

While designing sterically demanding ligands for Ln SMMs, alkoxides and aryloxides can be preferable over amides, alkyls, carbenes, silyls and many other softer ligands often used in the literature, even though these can bear a larger number of R groups. This is due to the hard Lewis basic O‐donor ligands being well‐matched with hard Lewis acidic and highly oxophilic Ln ions, as well as the ease of tunability of alkoxide and aryloxide electronic and steric properties.^[20]^ Electronic and steric saturation of Ln ions by alkoxides and aryloxides can provide relatively air‐ and temperature‐stable complexes, even in low CN environments.

**Scheme 1:**
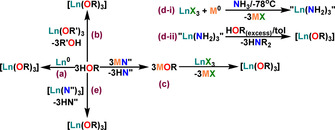
Synthetic strategies (a–e) towards Ln alkoxides and aryloxides; Ln=lanthanide, R=alkyl or aryl group; X=Cl, Br, I; M=Na, K, Li; N′′={N(SiMe_3_)_2_}; tol=toluene.

Although the term alkoxides was introduced in the 1840s,^[47]^ the chemistry of alkoxides was not well‐developed until the 1950s when Bradley and Wardlaw showed applications of alkoxides in various fields like catalysis, ceramic preparation via sol‐gel chemistry and MOCVD (Metal‐organic chemical vapour‐phase deposition).[^21^, ^22^, ^31^, ^48^, ^49^] Consequently, by the 1980s alkoxides of almost all the naturally occurring elements in the Periodic Table had been explored.[^21^, ^22^, ^31^, ^48^, ^49^] Various methods of synthesising alkoxides have been developed and are well‐established in the literature.[^20^, ^21^, ^22^, ^48^] Simple alkoxide and aryloxide group 1 ligand transfer agents are both economical and straightforward to prepare by reacting their corresponding alcohols with alkali metal or alkali metal hydrides.^[21]^ The synthesis and coordination chemistry of Ln alkoxides and aryloxides is discussed here (Scheme 1). The critical differences amongst the various methods of forming Ln alkoxide or aryloxide complexes come from the nature of the Ln starting material, such as metallic precursors, molecular halides, amides, alkoxides, hydrides and carboxylates, and organometallic species.[^20^, ^50^, ^51^]

Metal alcoholysis (Scheme 1 a) can be one of the most straightforward ways of producing Ln alkoxides and aryloxides, but the direct reaction of Ln metals (Ln^0^) and alcohols (HOR) with the evolution of H_2_ gas as an entropic driving force remains poorly‐developed due to the formation of oxide layers on Ln metals. Other techniques have been introduced in the literature[^52^, ^53^, ^54^, ^55^, ^56^] such as using HgCl_2_, Hg(C_6_F_5_)_2_ or liquid ammonia synthesis to clean and activate the metal surface. However, limitations like high sensitivity to water and difficulties in drying the alcohols have limited this method to making various molecular clusters due to the formation of alkoxy, oxo or hydroxo bridges.[^24^, ^25^, ^26^, ^57^, ^58^, ^59^, ^60^, ^61^]

Alcoholysis of simple Ln alkoxides by replacing the coordinated alkoxide ligands with other alcohols of interest (Scheme 1 b) is another way of synthesising desirable Ln alkoxides. This is helpful when the precursor is economical, easy to prepare, or commercially available. However, several factors like the solubility of the precursor in a product‐favourable solvent and similar enthalpies of formation of the starting material and product often lead to incomplete ligand exchange. This leads to the establishment of an equilibrium, which limits the purification and characterisation of products, rendering this method unfavourable to the synthesis of Ln alkoxide and aryloxide SMMs.

Salt metathesis is another route to produce Ln alkoxide and aryloxides and has been one of the most utilised methods to date. This technique commonly uses Ln halide precursors LnX_3_ (X=Cl, Br, I) or their THF adducts, and alkali metal alkoxides (Scheme 1 c). The enthalpic driving force of these reactions is the formation of insoluble alkali metal halide salts of relatively high lattice energies. The limitation here is the poor solubility of Ln halide precursors in less polar organic solvents and the facile formation of oxo‐bridges.[^24^, ^57^, ^62^]

Other methodologies exist in the literature to tackle the solubility issues of LnX_3_ precursors, such as performing an alkali metal reduction in anhydrous ammonia to give Ln tri‐amide intermediates, (Scheme 1 d‐i), which can undergo alcoholysis reactions with ROH (Scheme 1 d‐ii).[^63^, ^64^] The synthesis of Ln alkoxides or aryloxides by protonolysis reactions from Ln alkyls or amides (Scheme 1 e), has by far proven to be the most convenient synthetic route to low CN complexes.^[8]^ The formation of for example, HN(SiMe_3_)_2_ as a by‐product of these reactions is an effective driving force by consideration of *p*K_a_ values and Ln−O versus Ln−N or Ln−C bond strengths.^[7]^ This method can produce pure crystalline products; the only significant drawback with this approach is the time consumed in going through additional synthetic steps to synthesise Ln alkyl or amide precursors, though some of these are commercially available.[^20^, ^49^]

**Figure 6:**
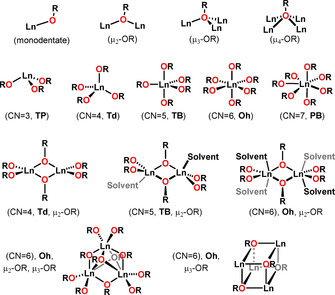
Bonding modes of alkoxide ligands and the coordination geometries commonly observed for Ln(OR)_*x*_ complexes. Top row: bonding modes of alkoxide ligands with varying bridging motifs. Second row, left to right: trigonal pyramidal (TP), tetrahedral (Td), trigonal bipyramidal (TB), octahedral (Oh), pentagonal bipyramidal (PB). Third row, left to right: Td, dinuclear; TB, dinuclear; Oh, dinuclear. Bottom row: Oh, multinuclear; the extra three bonds per vertex in the cube‐shaped bottom right structure are omitted for visual clarity.

The coordination chemistry of alkoxides and aryloxides strongly depends upon the host metal cation and the steric bulk of the ligand. Most of the Ln alkoxides and aryloxides found in the literature are either molecular clusters or poly‐nuclear bridged rings due to a combination of large Ln ionic size and small ligand R groups. Figure 6 summarises the common coordination geometries of Ln alkoxide and aryloxide mono and multinuclear complexes.

**Figure 7:**
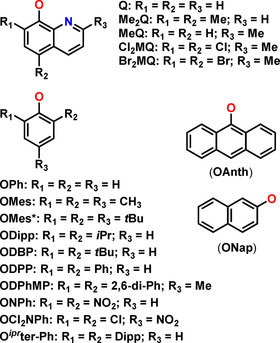
Selection of common aryloxide ligands in Ln chemistry.

The ability to easily change the substituents on the aromatic ring in aryloxides opens up a wide range of steric control. The Ln alkoxide literature is awash with examples of poly‐nuclear complexes from reactions that were intended to produce mono‐nuclear complexes.[^11^, ^20^, ^49^] Functionalisation of the parent aromatic ring in aryloxide ligands can provide the necessary steric bulk to prevent oligomerisation and can also change the ligand donor strength, which depends upon the electronic effects of substituents.^[20]^ The ability to make analogous complexes with differences in *ortho*‐, *meta*‐ and *para*‐substitution patterns can deliver interesting insights into the reactivity, physical properties, and control over metal coordination spheres. A wide range of aryl group substitution patterns exist in the literature for aryloxide ligands;[^15^, ^20^, ^65^, ^66^] for example the extensively used 2,6‐disubstituted phenoxides such as dimethylphenoxide (ODMP), di‐*iso*‐propylphenoxide (ODipp), di‐*tert*‐butylphenoxide (ODBP), and diphenylphenoxide (ODPP); 2,4,6‐trisubstituted phenoxides such as trimethylphenoxide (OMes), tris‐*tert*‐butylphenoxide (OMes*), and *o*‐bisdiphenyl‐*p*‐methylphenoxide (ODPhMP); and other derivatives such as quinilinolates (Q), anthroxides (OAnth) and napthoxides (ONap) (Figure 7).

**Figure 8:**
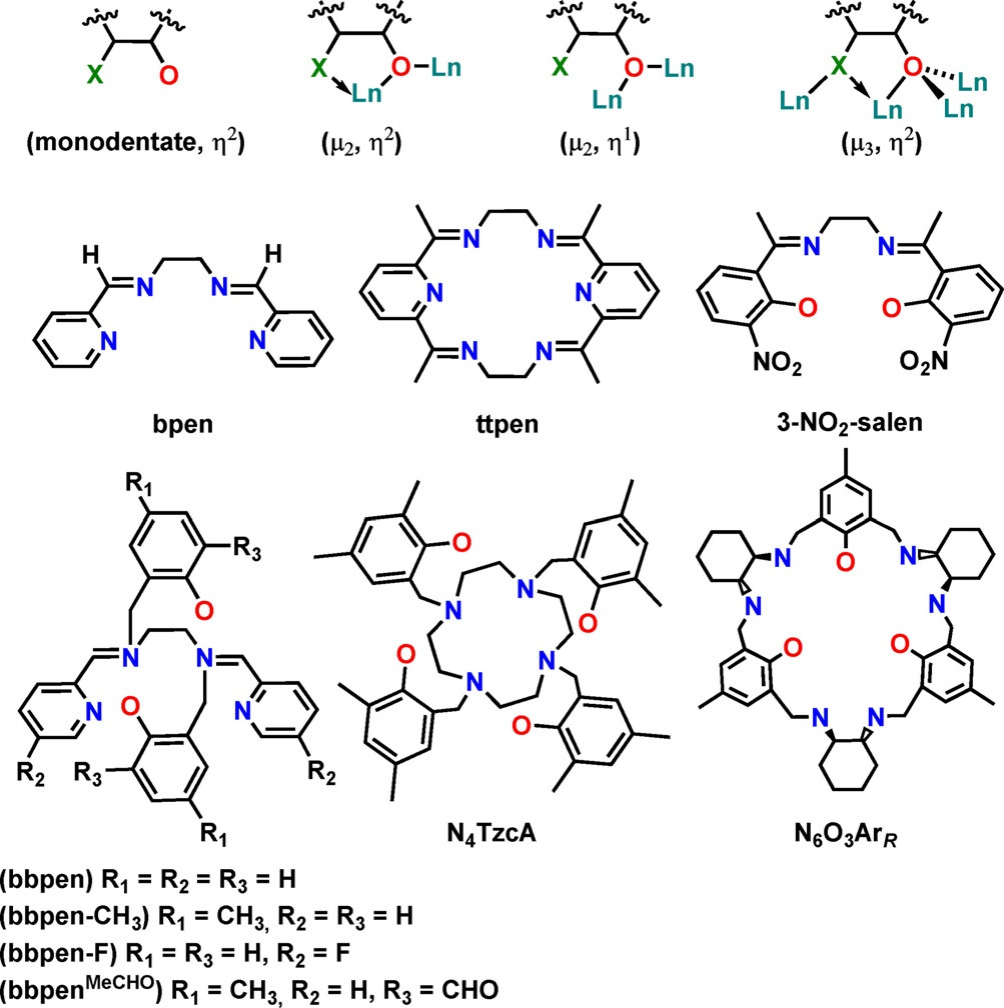
Bonding modes of donor functionalised chelating ligands (top row; Ln=lanthanide, X=N, P, S, etc.). Examples of large chelating ligands used in Dy alkoxide and aryloxide SMM chemistry (central and bottom row).

**Figure 9:**
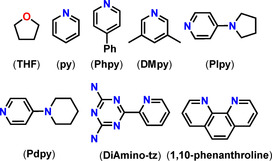
Various coordinating Lewis basic co‐ligands of relevance here.

From the Ln SMM design point of view, the potential multinuclearity promoted by Ln alkoxide and aryloxide complexes via various bridging modes (Figure 6 and Figure 8, 1st row) can be an issue. Other than using bulkier R groups, this challenge has often been tackled via chelating ligands (Figure 8, 2^nd^ and 3^rd^ row) or by the additional coordination of various neutral Lewis basic co‐ligands (e.g., NH_3_, THF, pyridine, etc.) Figure 9.[^12^, ^13^, ^15^]

## 5 Mononuclear Dy Alkoxide and Aryloxide SMMs

Table 1 lists mononuclear Dy^III^ SMMs that contain various mono‐ and multi‐dentate alkoxide and aryloxide ligands that have been structurally characterised to date. The complexes have been subdivided according to their formal CN, to highlight examples of higher CN numbers being more numerous. The *U*_eff_ values (rounded to whole numbers) and Dy−O distances (in Å) are listed for all complexes.

**Table 1:** Mononuclear Dy alkoxide and aryloxide SMMs, together with coordination number (CN), *U*_eff_, and Dy−O bond distances to alkoxide/aryloxide groups. Lattice solvents are omitted from the formulae. All ligand abbreviations are defined in the Appendix.

	Molecular Formula	*U*_eff_ [K]	Dy‐O_alk‐/aryl‐oxide_ [Å]	Ref.
**CN3**				
**3.1**	[Dy(O^*i*Pr^ter‐Ph)(O^*i*Pr^ter‐Ph′′)]	961	2.126(4)–2.191(4)	[65]
**CN4**				
**4.1**	[Dy(NPh_2_)(OCPh_3_)(μ‐OCPh_3_)_2_Li(THF)]	36	2.068(3)–2.273(4)	[67]
**CN5**				
**5.1**	[Dy(OMes*)_2_(THF)_2_Cl]	1262(32)	2.124(3)–2.121(3)	[15]
**5.2**	[Dy(OMes*)_2_(THF)_2_Br]	1210(10)	2.116(3)–2.120(3)	[15]
**5.3**	[Dy(OMes*)_2_(THF)_2_I]	1229(64)	2.124(3)	[15]
**CN6**				
**6.1**	[Dy(O*t*Bu)_2_(Phpy)_4_][BPh_4_]	2075(11)	2.066(8)	[41]
**6.2**	[Dy(O*t*Bu)_2_(Pdpy)_4_][BPh_4_]	1886(9)	2.110(4)–2.148(5)	[41]
**6.3**	[Dy(O*t*Bu)_2_(Plpy)_4_][BPh_4_]	1810(5)	2.136(5)–2.140(5)	[41]
**6.4**	[Dy(L^CO^){N(SiMe_3_)_2_}_2_]	190/262 @ 1000 Oe	2.4680(17)	[68]
**6.5**	[Dy(Me_2_Q)_2_Cl_3_(H_2_O)]	1110(50)	2.150(4)	[69]
**6.6**	[Dy(ODPhMP)(THF)_3_Cl_2_]	52(4)/52(4) @ 1400 Oe	2.088(3)	[70]
**6.7**	[Dy(Br_2_MQ)_2_(CH_3_CH_2_OH)Cl_3_]	37(1)/51(1) @ 2000 Oe	2.193(6)	[71]
**6.8**	[Dy(OAnth)_3_(py)_3_]	43@1000 Oe	2.131(3)–2.164(3)	[72]
**6.9**	[Dy(OCPh_3_)_2_(THF)_4_][BPh_4_]	1992(40)	2.103(1)	[73]
**CN7**				
**7.1**	[Dy(O*t*Bu)_2_(py)_5_][BPh_4_]	1815	2.110(2)–2.114(2)	[13]
**7.2**	[Dy(O*t*Bu)(THF)_5_Cl][BPh_4_]	938(150)	2.043(4)	[14]
**7.3**	[Dy(O*t*Bu)(THF)_5_Br][BPh_4_]	818(180)	2.023(4)	[14]
**7.4**	[Dy(OPh)(THF)_5_Cl][BPh_4_]	737(46)	2.113(4)	[14]
**7.5**	[Dy(OPh)_2_(THF)_5_][BPh_4_]	1329(112)	2.123(3)	[14]
**7.6**	[Dy(OPh)_2_(py)_5_][BPh_4_]	1302(45)/1197(26)^[a]^	2.1222(41)–2.1226(40)	[14]
**7.7**	[Dy(OEtPh)_2_(py)_5_][BPh_4_]‐Λ	1625(28)	2.109(3)–2.110(4)	[16]
**7.8**	[Dy(OEtPh)_2_(py)_5_][BPh_4_]‐Δ	1625(28)	2.089(4)–2.116(4)	[16]
**7.9**	[Dy(bbpen)Cl]	708(26)	2.166(4)	[12]
**7.10**	[Dy(bbpen)Br]	1025(17)	2.163(3)	[12]
**7.11**	[Dy(bpen)Cl(Cl_2_NphO)_2_]	86	2.616(3)–2.174(4)	[74]
**7.12**	[Dy(bpen)(Cl_2_NphO)_3_]	34	2.203(2)–2.187(2)	[74]
**7.13**	[Dy(bpen)(NphO)_3_]	27	2.156(3)–2.1935(19)	[74]
**7.14**	[Dy(bbpen‐CH_3_)Cl]	723	2.155(3)–2.166(3)	[75]
**7.15**	[Dy(bbpen‐CH_3_)Br]	1162	2.141(6)–2.151(7)	[75]
**7.16**	[Dy(bbpen‐F)Cl]	838	2.160(2)	[76]
**7.17**	[Dy(bbpen‐F)Br]	1150	2.155(2)	[76]
**7.18**	[DyCl_3_(HLQ^CO^)]	36 @ 700 Oe	2.408(2)–2.322(2)	[77]
**7.19**	[Dy(HN_4_TzcA)]	10 @ 2000 Oe	2.125(3)–2.199(3)	[78]
**7.20**	[Dy(H_4_N_6_O_3_Ar_*S*_)(SCN)_2_](SCN)_2_	–	2.202(9)–2.245(14)	[79]
**7.21**	[Dy(H_4_N_6_O_3_Ar_*R*_)(SCN)_2_](SCN)_2_	35 @ 200 Oe	2.194(10)–2.239(10)	[79]
**CN8**				
**8.1**	[Dy(ttpen)(OC_6_H_3_‐*t*Bu_2_‐2,4)_2_][PF_6_]	973	2.1302(14)–2.1456(14)	[17]
**8.2**	[Dy(tta)_2_(pyQ)]	68	2.281(2)–2.382(2)	[80]
**8.3**	[Dy(bbpen^MeCHO^)(tmpd)]	96	2.259(11)–2.369(12)	[81]
**8.4**	[Dy(acac)(ClNPhQ)_3_]	70/108^[a]^	2.276(11)‐2.332(11)	[82]
**8.5**	[Dy(DiAmino‐tz)_2_(salicylaldehyde)_2_]**⋅**Br	80	2.237(7)–2.366(7)	[83]
**8.6**	[Dy(DiAmino‐tz)_2_(salicylaldehyde)_2_]**⋅**Cl	112	2.242(6)–2.369(6)	[83]
**8.7**	[Dy(DiAmino‐tz)_2_(salicylaldehyde)_2_]**⋅**OH	168	2.203(5)–2.365(5)	[83]
**8.8**	[Dy(MQ)_2_(DiAmino‐tz)_2_]**⋅**Br	80	2.214(3)–2.217(3)	[84]
**8.9**	[Et_3_NH][Dy(3‐NO_2_‐salen)_2_]	40 @ 1500 Oe	2.260(6)–2.319(6)	[85]
**8.10**	[NMe_4_][Dy(dsp)_2_]	29 @ 500 Oe	2.239(8)–2.282(9)	[86]
**8.11**	[Dy(bbpen^MeCHO^)(Dppd)]**⋅**CH_3_OH**⋅**H_2_O	221/209	2.223(3)–2.267(3)	[87]
**8.12**	[Dy(bbpen^MeCHO^)(Dppd)]	279/244	2.219(4)–2.302(3)	[87]
**8.13**	[Dy(H_2_L1)_2_(CH_3_OH)_2_]**⋅**Cl	–	2.213(12)–2.379(12)	[88]
**8.14**	[Dy(H_3_L2)_2_(CH_3_OH)_2_]**⋅**Cl	–	2.247(15)–2.411(5)	[88]
**8.15**	[Dy(Br_2_MQ)_2_(1,10‐phenanthroline)(NO_3_)]	90 @ 1500 Oe	2.201(3)–2.209(3)	[89]
**8.16**	[Dy(Cl_2_MQ)_2_(1,10‐phenanthroline)(NO_3_)]	93 @ 1500 Oe	2.201(2)–2.202(3)	[89]
**8.17**	[Dy(Cl_2_MQ)_4_][Et_3_NH]	138 @ 2000 Oe	2.244(4)–284(5)	[90]
**8.18**	[Dy(Br_2_MQ)_4_][Et_3_NH]	76	2.245(4)–2.282(4)	[90]
**8.19**	[Dy(ODBquPh)_2_(THF)_2_][BPh_4_]	378(12)	2.190(6)	[91]
**8.20**	[Dy(ODBpyPh)_2_(py)_2_][BPh_4_]	389(12)	2.193(12)–2.196(12)	[91]
**8.21**	[Dy(OAr^8.21^)(hfac)_3_]	24(1) @ 1000 Oe	2.249(4)	[92]
**8.22**	[Dy(OAr^8.22^)(OTf)_2_(H_2_O)2][OTf]	33 @ 1200 Oe	2.216(4)	[93]
**8.23**	[Et_3_NH][Dy((R,R)/(S,S)‐3‐NO_2_salen)_2_]	40 @ 1500 Oe/18 @ 200 Oe	2.236(12)–2.327(12)	[94]
**8.24**	[Dy(ONpyPh)(tfa)_2_]	5 @ 2000 Oe	2.296(3)–2.419(3)	[95]
**8.25**	[Dy(HONpyPh)_2_]**⋅**Cl	44(1) @ 1000 Oe	2.292(6)–2.438(7)	[95]
**8.26**	[Dy(Cl_2_MQ)_3_(CH_3_CH_2_OH)(H_2_O)]	15 @ 1000 Oe	2.240(5)–2.291(6)	[96]
**8.27**	[Dy(bbpen)(tpe‐COOH)]	77 @ 1500 Oe	2.183(17)–2.216(17)	[97]
**8.28**	[Dy(bbpen)(OPPh_3_)_2_][BPh_4_]	944(28)	2.208(3)	[98]
**8.29**	[Dy(nmQ)(NO_3_)_2_(DMSO)][ClO_4_]	67 @ 2000 Oe	2.260(3)–2.92(3)	[99]
**8.30**	[Dy(DiAmino‐tz)_2_(o‐vanilin)_2_]**⋅**Br	221	2.215(6)–2.216(6)	[100]
**8.31**	[Dy(DiAmino‐tz)_2_(o‐vanilin)_2_]**⋅**NO_3_	615	2.238(4)–2.254(6)	[100]
**8.32**	[Dy(DiAmino‐tz)_2_(o‐vanilin)_2_]⋅CF_3_SO_3_	120	2.207(6)	[100]
**8.33**	[Dy(L_MC_)(hfac)_2_]**⋅**I	216	2.219(3)	[101]
**8.34**	[NEt_4_][Dy(Cl_2_Q)_4_]	–	2.269(2)–2.295(3)	[102]
**8.35**	[Dy(RhQ)(tta)_2_]	20(1) @ 1000 Oe	2.273(3)	[103]

[a] Two relaxation processes were observed.

### 5.1 Overview

From Table 1, the immediate observations are:

1): There are few mononuclear Dy alkoxide and aryloxide SMMs with CNs lower than seven.
2): The presence of Lewis basic donor solvents (e.g., THF and pyridine) influence both the nuclearity and coordination environment within these molecules.
3): Mononuclear Dy^III^ alkoxide and aryloxide complexes usually show coordination geometries described as trigonal pyramidal (TP), tetrahedral (Td), trigonal bipyramidal (TB), octahedral (Oh), pentagonal bipyramidal (PB) and hexagonal bipyramidal (HB). Ideal axially elongated‐Oh, PB and HB geometries would generate *D*_4*h*_, *D*_5*h*_ and *D*_6*h*_ local symmetries, respectively.[^13^, ^17^, ^41^] If these ideal local symmetries can be achieved they can create highly axial ligand fields and increase *U*_eff_ by restricting some degrees of freedom responsible for generating the transverse CF (eliminating $B_{k}^{q\neq 0}$ terms) thereby suppressing QTM.[^13^, ^17^, ^35^, ^41^] Unfortunately no molecules have been reported that have such ideal symmetry, and indeed strictly *D*_5*h*_ symmetry cannot be found in a crystalline material.
4): The number of geometries and topological variances decrease upon reducing the CN.^[67]^ The lower CN SMMs also tend to be sensitive towards air and moisture.[^15^, ^65^, ^67^]
5): *Ortho*‐substituted bulky phenoxide ligands can provide sufficient steric crowding to form low‐coordinate mono‐nuclear Dy aryloxide complexes,[^15^, ^65^] hence introducing bulkier, as well as electron‐donating groups such as *tert*‐butyl and *iso*‐propyl at the *ortho*‐ and *para*‐positions of aryloxides, can be beneficial in realising target low‐coordinate mono‐nuclear Dy complexes.

The respective complexes of various CN with their notable attributes are discussed below.

### 5.2 CN 3, 4 and 5 complexes

**Figure 10:**
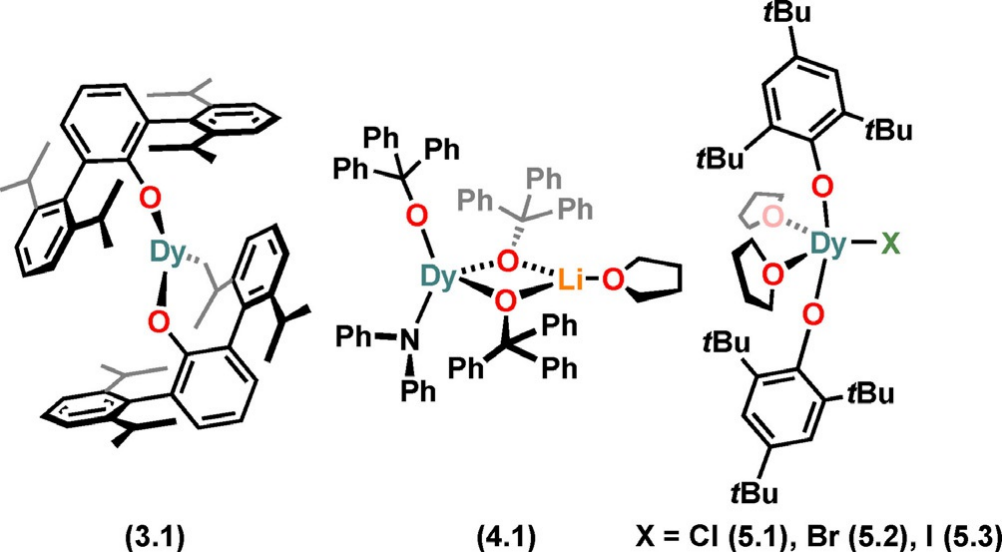
Molecular structures of mononuclear Dy^III^ alkoxide and aryloxide SMMs of CN 3–5.

Complex **3.1** is the only three coordinate mononuclear Dy aryloxide SMM that has been reported to date.^[65]^ This complex utilises the bulky terphenyl ligand O^*i*Pr^ter‐Ph (OC_6_H_3_Dipp_2_‐2,6) to restrict the coordination sphere of the Dy^III^ centre (Figure 10). Complex **3.1** was synthesised via protonolysis of [Dy{HC(SiMe_3_)_2_}_3_] by two equivalents of the terphenol proligand. In the solid‐state structure of **3.1**, two of the donor atoms to Dy are the oxygen atoms of the two terphenoxide ligands, with the coordination sphere of Dy completed by a Dy−C bond that arises from C−H bond activation of one of the terphenoxide ligands. The Dy−O bond distances of **3.1** are 2.191(4) and 2.126(4) Å and the short Dy‐C distance is 2.54(1) Å. The _Ar_O‐Dy‐O_Ar_ angle of **3.1** is 144.3(1)°.^[65]^

Complex **4.1** is the only four‐coordinate mononuclear Dy^III^ alkoxide SMM reported to date.^[67]^ The Dy^III^ centre in **4.1** is in a distorted Td geometry with one anionic NPh_2_, one terminal Ph_3_CO and two μ_2_‐Ph_3_CO bridging to a Li(THF) fragment (Figure 10). This complex has one of the shortest Dy−O_terminal_ distances (2.068(3) Å) amongst Dy^III^ alkoxide and aryloxide SMMs. It was synthesised via a salt metathesis reaction of DyCl_3_ with lithiated amide/alkoxide ligand transfer agents in THF.^[67]^

There is only one family of five‐coordinate Dy aryloxide SMMs in the literature.^[15]^ Complexes **5.1–5.3** contain Dy^III^ centres in distorted square‐based pyramidal geometries. The degree of distortion (τ_5_) within the structural continuum between square‐ based pyramidal (τ_5_=0) and trigonal bipyramidal (τ_5_=1) for complexes **5.1–5.3**, was found to be 0.348, 0.344 and 0.340, respectively.^[15]^ The base of the pyramid contains the two aryloxide ligands *trans*‐ to each other, while two THF molecules form the other *trans*‐ pair, with a halide at the apex (Figure 10).^[15]^ These complexes were synthesised via salt metathesis methods, by reacting THF adducts of DyX_3_ {X=Cl (**5.1**), Br (**5.2**), I (**5.3**)} with two equivalents of MOMes* {M=Na (**5.1**), K (**5.2, 5.3**)} in THF at room temperature. The Dy−O_Ar_ distances of **5.1–5.3** range from 2.116(3) to 2.124(3) Å and the _Ar_O‐Dy‐O_Ar_ angles span from 146.4(1) to 148.3(2)°.^[15]^

### 5.3 CN 6 complexes

**Figure 11:**
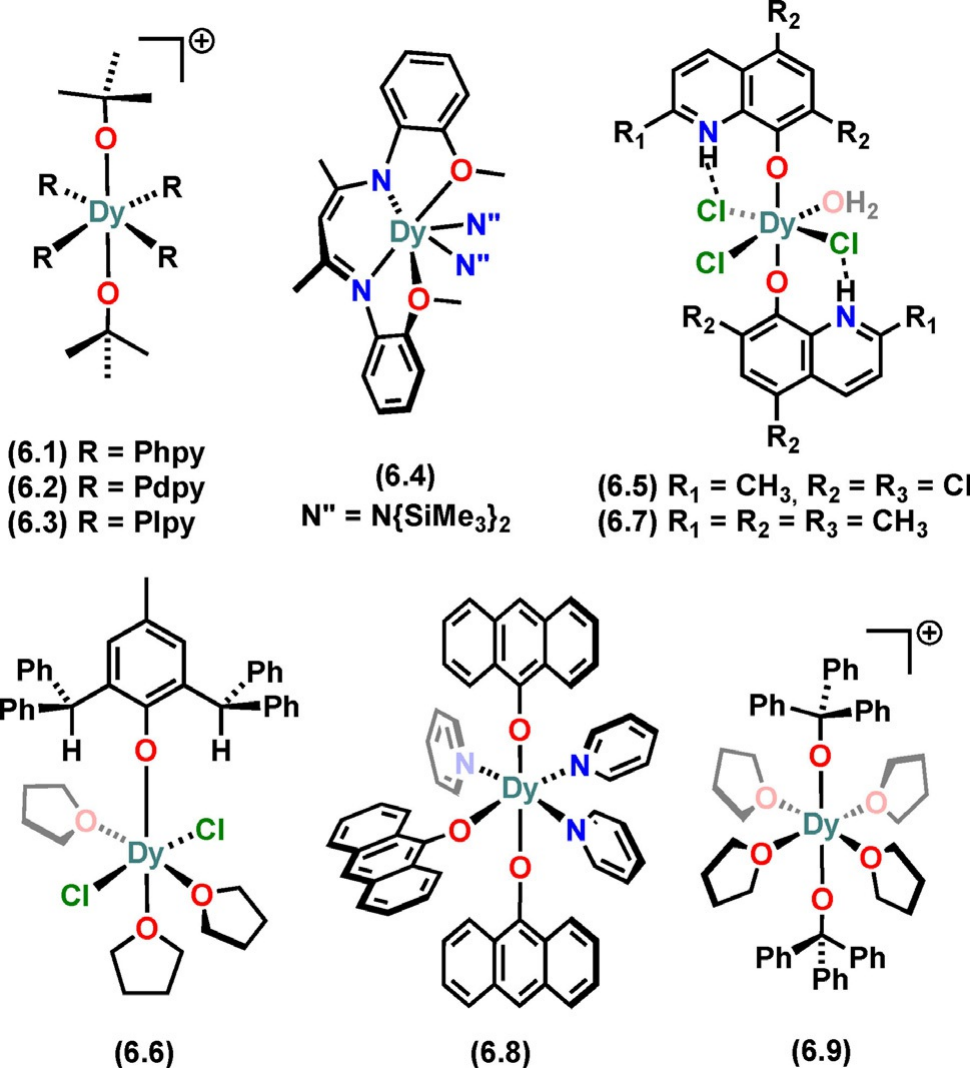
Molecular structures of mononuclear Dy^III^ alkoxide and aryloxide SMMs of CN 6. Counter‐ions not shown for brevity.

There are only a handful of six‐coordinate mononuclear Dy alkoxide and aryloxide SMMs in the literature.^[41]^ The Dy^III^ ions in **6.1–6.3** have octahedral geometries with two mutually *trans*‐alkoxides and four equivalent neutral co‐ligands at the equatorial positions, along with a BPh_4_^−^ counter anion for charge balance (Figure 11).^[41]^ These complexes were synthesised via salt metathesis protocols by refluxing DyCl_3_ with 2NaO*t*Bu and NaBPh_4_ in THF overnight, then adding six equivalents of respective co‐ligands (Phpy (**6.1**), Pdpy (**6.2**), Plpy (**6.3**)) after concentrating the THF solution to saturation.^[41]^ The O‐Dy‐O angles for **6.1–6.3** are in the range 178.9(2) to 180°, and the Dy‐O distances range from 2.066(8) to 2.148(5) Å.

Complex **6.4** was reported by Liu et al. in 2017,^[68]^ and has the Dy^III^ centre in a trigonal prismatic geometry (Figure 11). It showed magnetic hysteresis up to 3 K in the absence of an external magnetic field, with a *U*_eff_ of 190 K. Giansiracusa *et al* reported **6.5** recently.^[69]^ This complex utilises methyl‐functionalised quinolinolate (Me_2_Q) ligand (Figure 7) *trans*‐ to each other in the complex, with the Dy^III^ centre in an approximately octahedral geometry with a perfectly linear O‐Dy‐O angle and three anionic chloride donors and a neutral H_2_O donor at the equatorial positions (Figure 11). Despite showing a high *U*_eff_ of 1110(50) K, **6.5** does not show magnetic hysteresis, even at 2 K. The *τ*_switch_ (timescales at which the Raman process takes over the Orbach process^[18]^) value of 1.36×10^−4^ s, which is the lowest known for any SMM with *U*_eff_>1000 K, suggests a dominant Raman relaxation process.^[69]^ Complex **6.6**, which contains an aryloxide (ODPhMP) ligand, exhibits Dy^III^ in a distorted Oh geometry (Figure 11) and does not show any remarkable SMM behaviour in the absence of external field, with a *U*_eff_ of 52(4) K at 0 as well as 1400 Oe applied dc field.^[70]^ Complexes **6.7** ^[71]^ and **6.8** ^[72]^ have recently been reported by Yin et al. and Long et al., respectively. Both complexes contain Dy^III^ centres in approximate Oh geometries (Figure 11) and show weak SMM behaviour, with *U*_eff_ of 37(1) K for **6.7** and 43 K @1000 Oe for **6.8**. Complex **6.9** is another mononuclear Oh Dy^III^ alkoxide SMM that has been recently reported in 2021 by Long et al.^[73]^ Complex **6.9** shows a high energy barrier of 1992(40) K, with luminescence behaviour also observed. Magnetic and computational analysis of **6.9** showed that as well as the high axiality arising from the *trans*‐ arrangement of alkoxide ligands, an unusual mechanism is responsible for the high *U*_eff_ value: the large energy gaps amongst the first three excited states surpass the available phonon energies, thereby suppressing the one‐phonon transitions between the three low‐lying CF multiplets, even in the absence of a perfectly axial CF.^[73]^

### 5.4 CN 7 complexes

**Figure 12:**
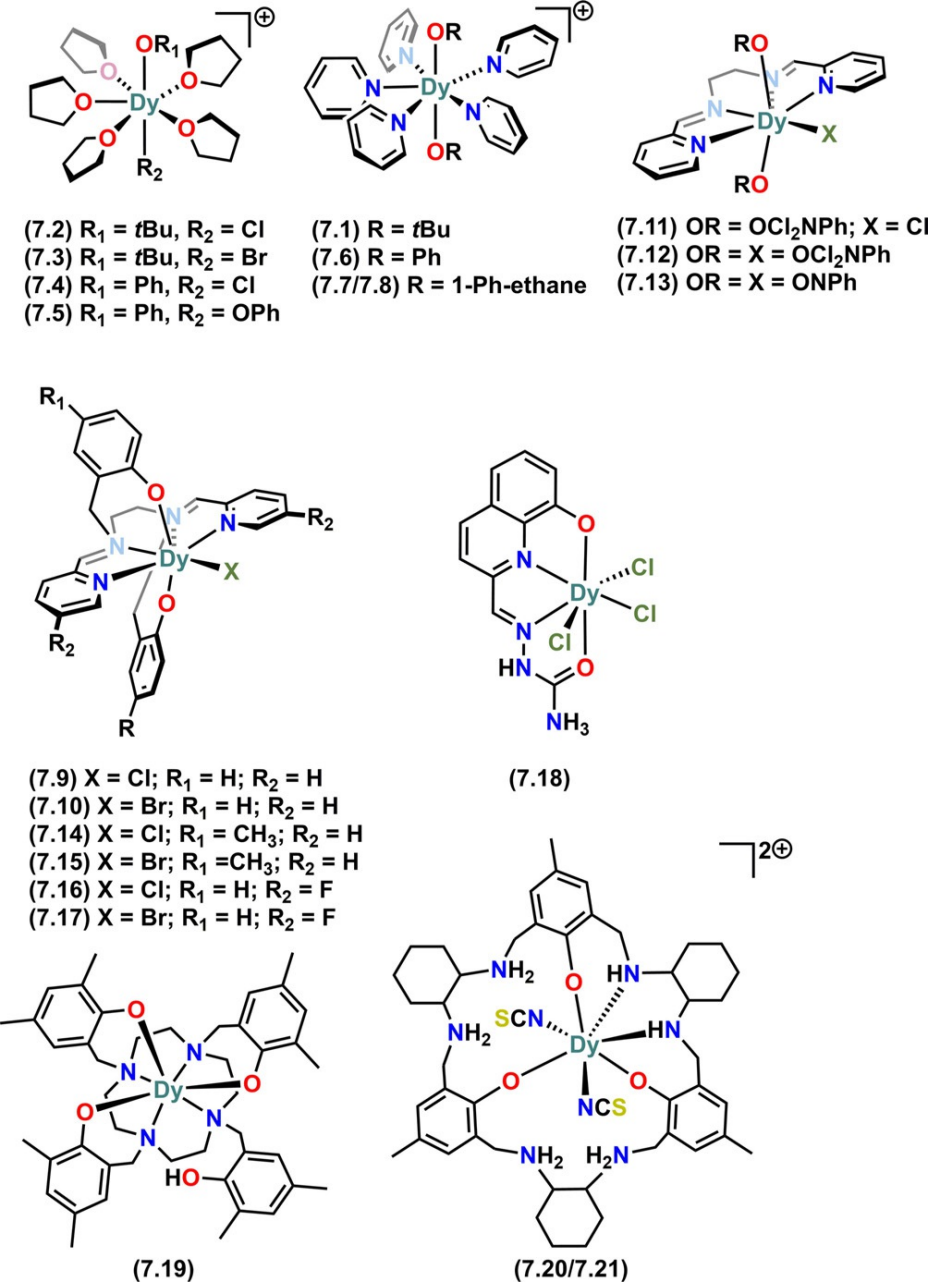
Molecular structures of mononuclear Dy^III^ alkoxide and aryloxide SMMs of CN 7. Counter‐ions not shown for brevity.

Due to the aforementioned recent enhanced interest in understanding pentagonal bipyramidal (PB) mononuclear Dy^III^ SMMs, there are now multiple seven‐coordinate Dy‐alkoxide and aryloxide SMMs in the literature (Figure 12).[^12^, ^13^, ^14^, ^75^, ^76^] The majority of these complexes have been synthesised by a common approach and have a PB geometry.[^14^, ^76^] Complexes **7.1–7.8** contain either one or two monodentate alkoxide or aryloxide ligands at the axial positions and Lewis basic monodentate neutral ligands at the five equatorial positions.[^14^, ^16^] The equatorial ligands in these complexes arise from the coordinating solvent used in the reaction mixture, saturating the coordination sphere about Dy to limit the nuclearity, which is further ensured by the weakly‐coordinating separated BPh_4_ counter‐anion.[^14^, ^16^]

Complexes **7.1, 7.5** and **7.6–7.8** were prepared by salt metathesis reactions of DyCl_3_ with two equivalents of NaOR (R=alkyl or aryl} and one equivalent of NaBPh_4_.[^14^, ^16^] Complexes **7.2–7.4** were prepared similarly but using only one equivalent of NaOR, hence they contain a halide at one axial position, *trans*‐ to OR.^[14]^ Complex **7.9** and its Br analogue **7.10** were discovered in 2016 and show a high energy barrier to magnetic reversal (1025 K).^[12]^ These complexes utilise a single large multi‐dentate dianionic ligand bbpen (Figure 12) to encapsulate the Dy^III^ centre, with a halide at one of the equatorial positions as a co‐ligand along with other *N*‐donors of the encapsulating bbpen ligand.^[12]^ The Dy−O distances in **7.1–7.10** range from 2.023(4) to 2.166(4) Å. The axial L1‐Dy‐L2 (L1=L2=O*t*Bu, OPh, O_bbpen_; L1=O*t*Bu, L2=Cl, Br; L1=OPh, L2=Cl, Br) angles range from 154(1)° to 179(1)°.

Complexes **7.11–7.17** contain various derivatives of bpen and bbpen ligands with different co‐ligands and are synthesised similarly to **7.9/7.10**.[^74^, ^75^, ^76^] Complexes **7.11–7.13** were reported by Li et al. for studying the variation of magnetic behaviour in PB Dy^III^ SMMs by varying individual co‐ligands alongside the main bpen ligand.^[74]^ Using a 4‐methyl functionalised bbpen ligand Jiang et al. reported **7.14** and **7.15** in 2018;^[75]^ these complexes are structurally similar to **7.9** and **7.10**, containing an electron‐donating methyl group at the 4‐position of the main anionic donating site (phenoxide on bbpen ligand). Another set of bbpen‐ functionalised complexes (**7.16** and **7.17**) were reported very recently by Zhu et al.^[76]^ The authors modified the bbpen ligand by introducing an electronegative F atom onto the equatorial donor site of the chelating bbpen ligand to synthesise **7.16** and **7.17**, which are analogous to **7.9** and **7.10**, respectively.^[76]^

Complex **7.18** contains a tetradentate neutral quinolinolate‐based LQ^CO^ ligand and was reported by Yang et al. in 2019.^[77]^ This complex has the Dy^III^ centre in a nearly ideal PB geometry with three anionic chloride donors in equatorial positions, and showed weak SMM behaviour, with a *U*_eff_ of 36 K.^[77]^ Using the large multi‐dentate N_4_TzcA ligand (Figure 8), Wen et al. reported a multifunctional SMM (**7.19** ^[78]^) recently, which contains a Dy^III^ centre with four *N*‐donors and three O‐donors showing field‐induced SMM behaviour as well as strong fluorescent emission.^[78]^ Complexes **7.20** and **7.21** are rare examples of chiral mononuclear seven‐coordinate SMMs containing large macrocyclic ligands (Figure 8).^[79]^ The Dy^III^ centres in these homochiral complexes sit in a saddle‐type conformation; **7.21** shows weak field‐induced SMM behaviour.^[79]^

### 5.5 CN 8 complexes

**Figure 13:**
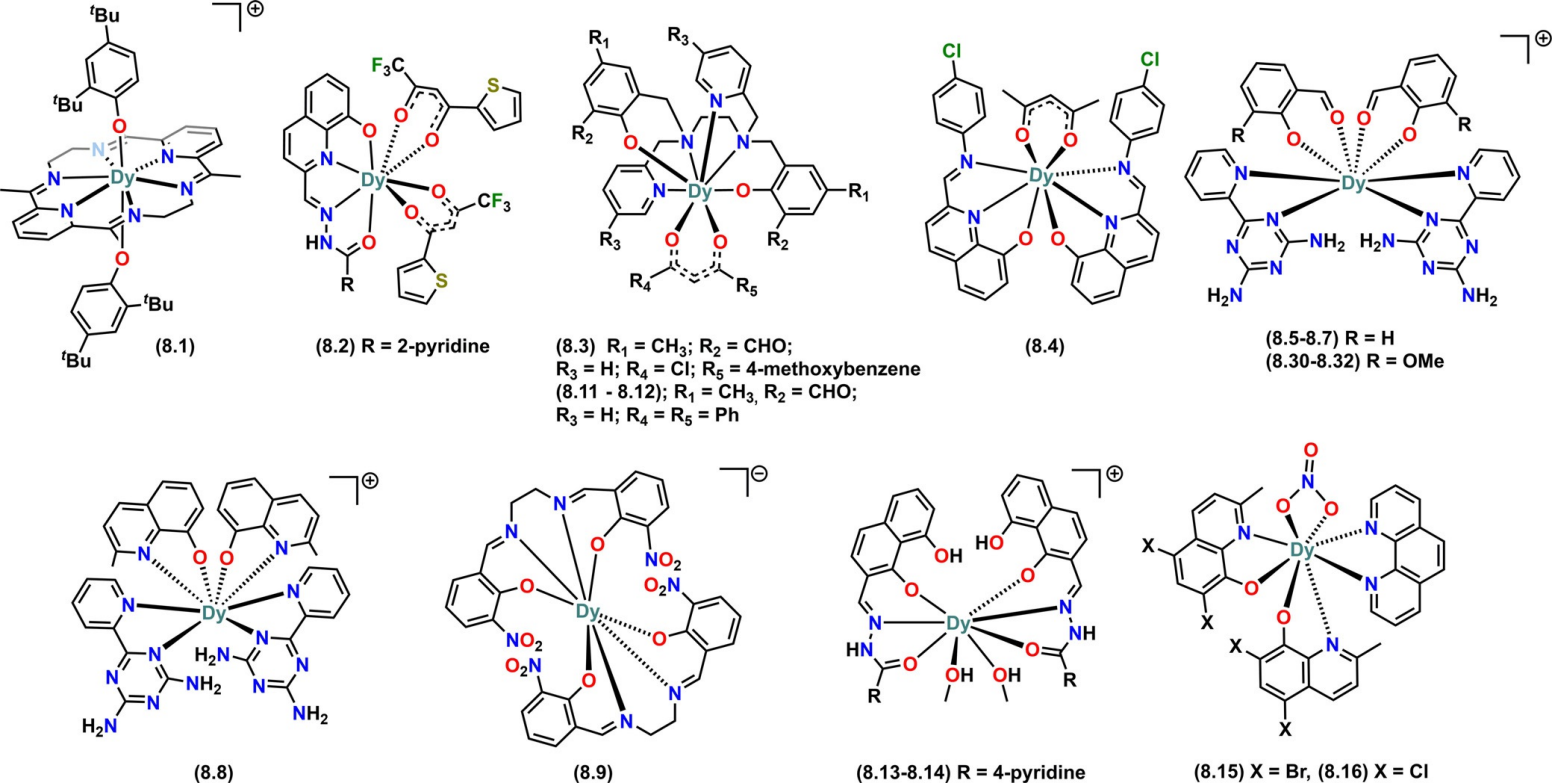
Molecular structures of mononuclear Dy^III^ alkoxide and aryloxide SMMs of CN 8 (part 1 of 3). Counter‐ions not shown for brevity.

Complex **8.1** was reported in 2019 and exhibits the highest *U*_eff_ (973 K) amongst mononuclear eight‐coordinate Dy^III^ alkoxide and aryloxide SMMs;^[17]^ it is also the only eight‐coordinate SMM in Table 1 that has two monodentate aryloxide ligands. The Dy^III^ site in complex **8.1** shows an approximate hexagonal bipyramidal geometry with the two aryloxide ligands (OC_6_H_3_‐*t*Bu_2_‐2,4; ODBP‐2,4) at the axial positions, with the six N‐donors from the bulky co‐ligand (ttpen) on the equatorial plane (Figure 13).^[17]^ The synthetic route to **8.1** is somewhat unusual for Ln alkoxide and aryloxides, being a two‐step process wherein the weakly coordinating ttpen ligand is coordinated firstly by reacting aqueous Dy^III^ acetate with 2,6‐diacetylpyridine and ethylenediamine (generating ttpen in situ) in methanol to give [Dy(ttpen)(CH_3_CO_2_)_2_](CH_3_CO_2_).^[17]^ This intermediate was then reacted with two equivalents of NaODBP‐2,4 in dichloromethane to replace the weak axial acetate ligands along with one equivalent of KPF_6_ to give **8.1**. The PF_6_ counter anion balances the +ve charge of the cation.

**Figure 14:**
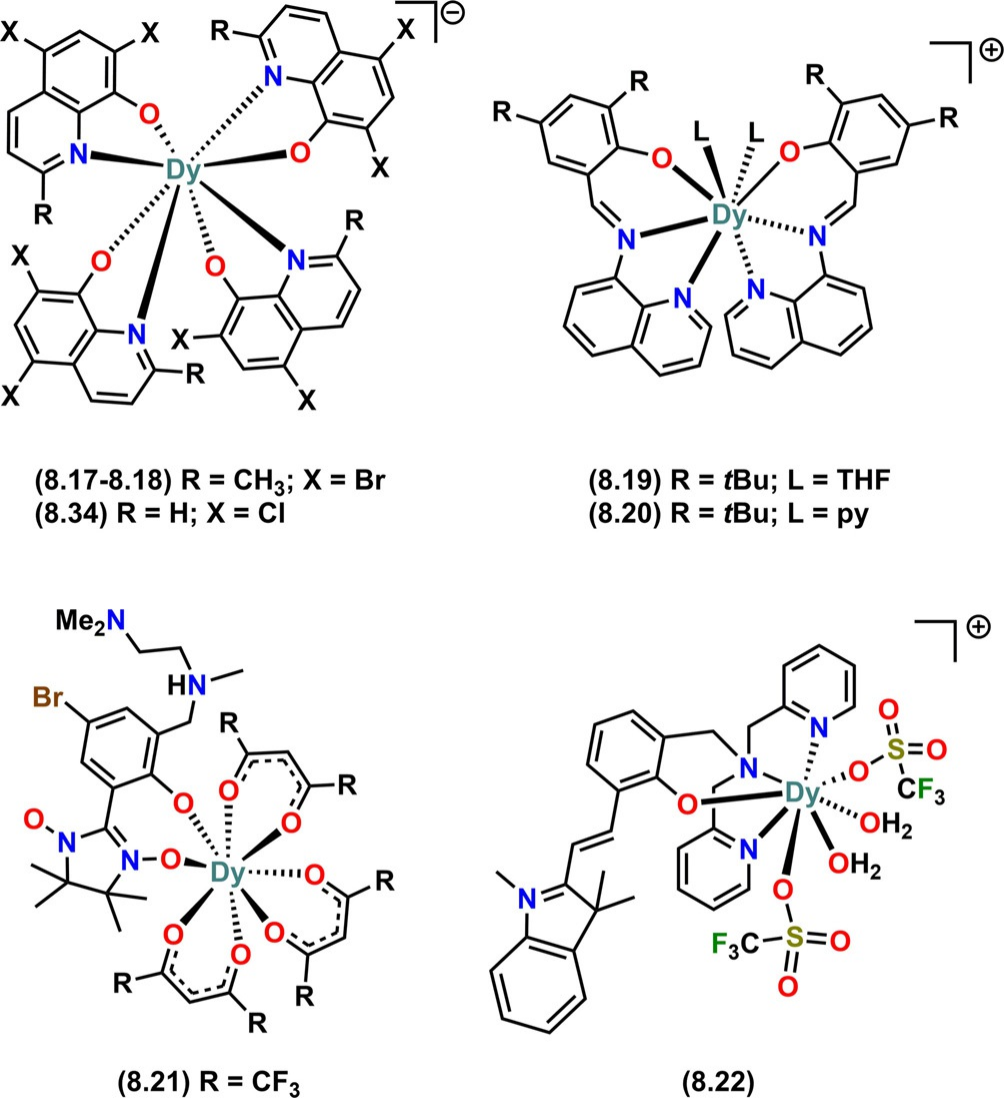
Molecular structures of mononuclear Dy^III^ alkoxide and aryloxide SMMs of CN 8 (part 2 of 3). Counter‐ions not shown for brevity.

The other eight‐coordinate SMMs of Table 1 were all reported within the last six years and contain multi‐dentate ligands such as the various functionalised quinolinolate derivatives in **8.17** and **8.18**,^[90]^ **8.29** ^[99]^ and **8.34**;^[102]^ (Figure 14) or functionalised quinolinolate derivatives with the following co‐ligands: (i) functionalised acetate in **8.2**,^[80]^ **8.4** ^[82]^ and **8.35**;^[103]^ (ii) nitrate in **8.15** and **8.16**;^[89]^ (iii) DiAmino‐tz in **8.8**;^[88]^ (iv) CH_3_OH in **8.13** and **8.14**;^[88]^ and, (v) H_2_O and EtOH in **8.26**.^[96]^ The average Dy‐O distances amongst the functionalised quinolinolate SMMs range from 2.213(12)–2.382(3) Å and the highest *U*_eff_ (138 K) is observed in **8.17**.^[90]^

**Figure 15:**
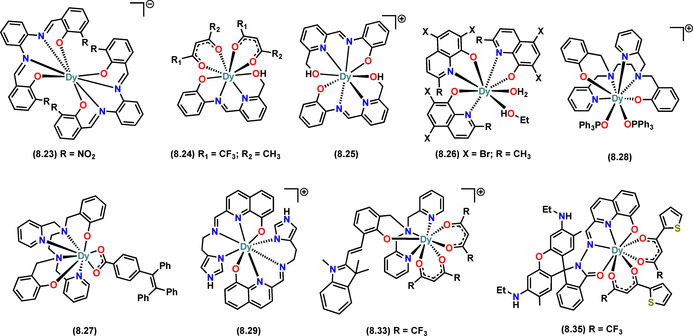
Molecular structures of mononuclear Dy^III^ alkoxide and aryloxide SMMs of CN 8 (part 3 of 3). Counter‐ions not shown for brevity.

Complexes **8.5–8.7** ^[83]^ and **8.30–8.32** ^[100]^ contain salicylaldehyde and 6‐methoxy‐salicylaldehyde as the main ligands, and DiAmino‐tz as co‐ligands, respectively. Complexes **8.30–8.32** showed modulation of magnetic relaxation behaviour via geometric isomerism.^[100]^ Using the bulkier Salen‐type ligands (Figure 8), Ren et al. reported **8.9** ^[85]^ and **8.23 (**Figure 15 **)**,^[94]^ which both contain DyN_4_O_4_ cores; **8.9** showed slow relaxation and photoluminescence,^[85]^ whilst homochiral **8.23** displayed a field‐induced double relaxation process.^[94]^

As can be seen in the CN7 complexes, bbpen is a versatile ligand. Indeed many CN8 SMMs exist with functionalised bbpen ligands and various co‐ligands such as: (i) tmpd in **8.3**;^[81]^ (ii) dppd in **8.11** and **8.12**;^[87]^ (iii) tpe‐COOH in **8.27**;^[97]^ and, (iv) OPPh_3_ in **8.28**;^[98]^ these were reported by Zheng et al. (**8.3**, **8.11** and **8.12**), and Tong and co‐workers (**8.27** and **8.28**). Complexes **8.11** and **8.12** have DyN_4_O_4_ cores with approximately trigonal dodecahedral configurations. Complex **8.11** can reversibly transform to **8.12** via a single‐crystal to single‐crystal (SCSC) transformation under different solvent environments, thus showing the influence of lattice solvent on the crystal structure, which the authors exploited to perform magneto‐structural correlations in **8.11** and **8.12**.^[87]^ Complex **8.27** was synthesised by replacing the Br from **7.10** with 4′‐(1,2,2‐triphenylvinyl)benzoic acid (tpe‐COOH); **8.27** shows weak field‐induced SMM behaviour, with a sharply decreased *U*_eff_ of 77 K compared to 1025 K in **7.10**.^[97]^ Complex **8.28** contains a Dy^III^ ion in a square antiprismatic geometry, and exhibits a remarkable *U*_eff_ of 944 K under no external field, with $T_{B}^{hyst}$ ≈6 K.^[98]^

## 6 Discussions of Selected High‐barrier Dy^III^ Alkoxide and Aryloxide SMMs

**Figure 16:**
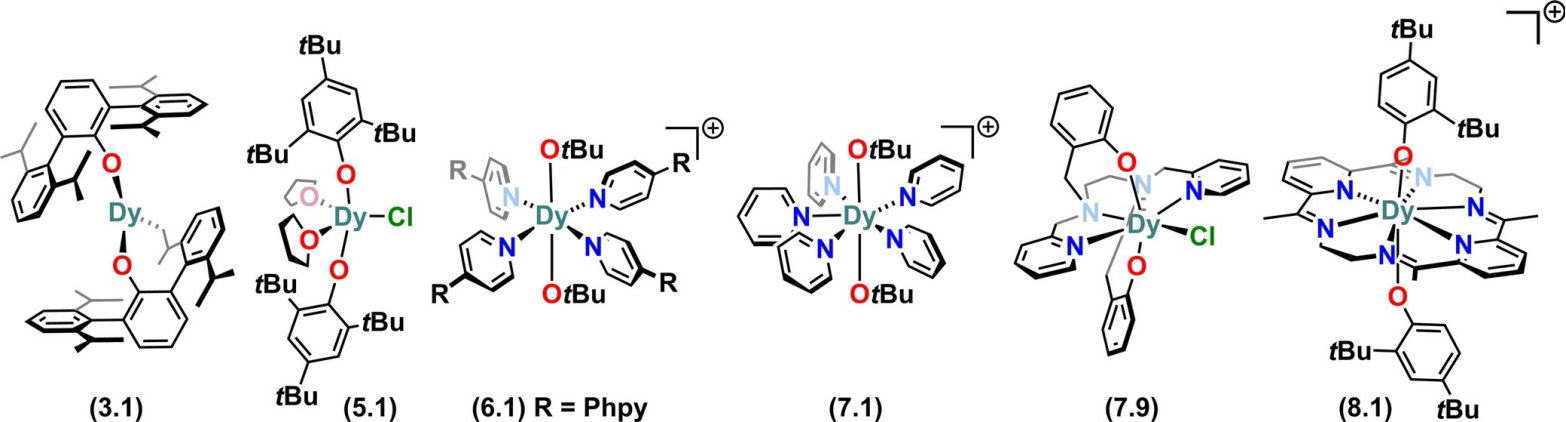
Molecular structures of selected SMMs from Table 2.

Table 2 and Figure 16 contain a selection of recent examples of mononuclear alkoxide and aryloxide SMMs with Dy^III^ centres from coordination numbers of 3 to 8 and highly axial ligand fields, giving large *U*_eff_ values. Table 2 also lists the important metrical parameters of the crystal structures that play a significant role in determining their SMM behaviour (Dy−O bond distances and the axial RO‐Dy‐OR angle), their energy barriers, and computational results of the electronic structure.

**Table 2:** Selected high‐performing Dy^III^ alkoxide and aryloxide SMMs.^[a]^

Complex	Systematic name	*U*_eff_ [K]	C [K^−*n*^ s^−1^]	*n*	THystB [K]	TZFCB [K]	Dy−O distance [Å]	RO‐Dy‐OR angle [°]	GS−ES1 [K]^[c]^	g_x_	g_y_	g_z_	∢g_z_	Ref.
**8.1**	[Dy(ttpen)(OC_6_H_3_ *t*Bu‐2,4)_2_][PF_6_]	973	0.37	2.5			2.130(1)–2.146(1)	176.54(5)	620	0.217	0.242	16.988	4.670	[17]
**7.1**	[Dy(O*t*Bu)_2_(py)_5_][BPh_4_]	1815	1.62×10^−6^	3.6(2)	4	14	2.110(2)–2.114(2)	178.91(9)	812	0	0	16.97	0.36	[13]
**7.2**	[Dy(O*t*Bu)(THF)_5_Cl][BPh_4_]	938(150)	1.77×10^−6^	4.7(15)	11	7	2.043(4)	178.26(9)	552	0.02	0.02	16.97	0.6	[14]
**7.3**	[Dy(O*t*Bu)(THF)_5_Br][BPh_4_]	818(180)	4.46×10^−5^	3.7(7)	9	4.5	2.023(4)	178.0(1)	495	0.01	0.01	16.99	0.6	[14]
**7.4**	[Dy(OPh)(THF)_5_Cl][BPh_4_]	737(46)	1.51×10^−5^	4.1(6)	9	6.8	2.113(4)	178.47(11)	495	0.05	0.06	16.88	0	[14]
**7.5**	[Dy(OPh_)_2(THF)_5_][BPh_4_]	1329(112)	1.02×10^−6^	4(1)	18	12	2.123(3)	176.34(10)	690	0	0	17	1.8	[14]
**7.6**	[Dy(OPh)_2_(py)_5_][BPh_4_]	1302(45)/1197(26)^[b]^	4.07×10^−6^/2.13×10^−8[b]^	4.2(2)/5.1(1)^[b]^	16	13	2.122(4)–2.123(4)	176.4(3)	688	0.01	0.01	16.98	0.6	[14]
**7.9**	[Dy(bbpen)Cl]	708(26)			8	7.5	2.166(4)	154.3(2)	550	0.11	0.16	16.91	0.50	[12]
**7.10**	[Dy(bbpen)Br]	1025(17)	1.49×10^−4^	3.5	14	9.5	2.163(3)	155.8(2)	567	0.06	0.07	16.98	0.52	[12]
**6.1**	[Dy(O*t*Bu)_2_(Phpy)_4_][BPh_4_]	2075(11)	5.60×10^−3^	2.85(4)			2.066(8)	180	926	0.01	0.01	17.28	0.20	[41]
**6.2**	[Dy(O*t*Bu)_2_(Pdp‐py)_4_][BPh_4_]	1886(9)	1.49×10^−3^	2.76(3)			2.110(4)–2.148(5)	178.90(15)	870	0.01	0.01	16.88	1.4	[41]
**6.3**	[Dy(O*t*Bu)_2_(Plpy)_4_][BPh_4_]	1810(5)	1.94×10^−3^	3.08(5)			2.136(5)–2.140(5)	180	828	0.02	0.02	17.18	1.7	[41]
**5.1**	[Dy(OMes*)_2_(THF)_2_Cl]	1262(32)	1.26×10^−2^	2.9(1)	5		2.124(3)–2.121(3)	146.4(1)	596	0.02	0.02	16.98	0.78	[15]
**5.2**	[Dy(OMes*)_2_(THF)_2_Br]	1210(10)	4.47×10^−3^	2.86(2)	5		2.116(3)–2.120(3)	148.3(1)	630	0.01	0.01	16.99	1.54	[15]
**5.3**	[Dy(OMes*)_2_(THF)_2_I]	1229(64)	3.80×10^−3^	2.42(4)	5		2.124(3)	148.3(2)	644	0.01	0.01	17	1.88	[15]
**3.1**	[Dy(O^*i*Pr^ter‐ph)(O^*i*Pr^ter‐ph′′)]	961			6	7	2.126–2.191	144.3(1)	547					[65]

[a] g_x,_ g_y,_ g_z_ are the calculated g factors of the first excited state (ES_1_); ∢g_z_ deviation of the g_z_ vector from the ground to the first excited state; OR=alkoxide and aryloxide ligands. [b] Two relaxation processes were observed. [c] GS−ES_1_=calculated magnetic ground state and first excited state energy gap.

The Dy‐O alkoxide and aryloxide bond distances vary considerably in the selection of complexes in Table 2, spanning from 2.02–2.14 Å; these are also sensitive to the nature of the co‐ligands. It is clear that the Dy−O distance is significantly affected by the CN and the donor strength of co‐ligands. In all the mononuclear Dy aryloxides and alkoxides in Table 2, the principal magnetic axis passes through or is aligned with the Dy−O bond. Hence, the Dy−O distance should also influence the CF effects governing the magnetic behaviour.

### 6.1 Effect of co‐ligands

Complex **6.1** ^[41]^ has one of the shortest Dy−O distances, a Dy‐O‐Dy angle of 180° and four weak equatorial donors; to date, this complex shows the highest *U*_eff_ value as well as the biggest GS‐ES_1_ energy gap for any Ln alkoxide or aryloxide SMM. However, having a high energy barrier is not the sole requirement to provide improved SMMs, and this is when the effects of co‐ligands can help. A lower number of co‐ligands at the equatorial positions will reduce the transverse CF components and will lower the probabilities of QTM. The donor strength of equatorial ligands affects both the *U*_eff_ and relaxation times by varying the probability of QTM. Complexes **5.1–5.3** ^[15]^ and **7.9** and **7.10** ^[12]^ represent two families of respective five‐ and seven‐coordinate SMMs that bear an anionic halide donor at the equatorial position relative to the principal magnetic axis. In both families, the SMMs performance improves when moving from the more strongly bound and charge‐dense Cl anion to heavier and more charge‐diffuse Br and I anions. From **5.1–5.3** ^[15]^ the $\tau_{QTM}$ increases from 0.066 s in Cl to 0.257 s in Br to 0.436 s in the I‐analogue, showing an ≈80 % decrease in QTM relaxation rates across this series. The GS‐ES_1_ gap also increases from 596 K in **5.1** to 644 K in **5.3**; such increases in GS‐ES_1_ gaps have been observed amongst all analogues within the same families of complexes in Table 2, where the donor strength of equatorial ligands has been reduced. Among a similar family of molecules, the effect of neutral co‐ligands on SMM behaviour was shown by Liu et al. recently.^[76]^ Complexes **7.16** and **7.17** ^[76]^ have a weaker electrostatic field on the equatorial plane of the Dy^III^ centre due to the bbpen ligand functionalisation by the electron‐withdrawing F atoms. Consequently, **7.16** and **7.17** show improved SMM properties (higher *U*_eff_ and $T_{B}^{Hyst}$ ) compared to **7.9** and **7.10** respectively.

Table 2 also shows the g_x_≈g_y_, and g_z_ values and the angle of deviation of the g_z_ vector from the principal magnetic axis of the first excited state (ES_1_). These values are generated by ab initio CASSCF‐SO calculations using the single‐crystal XRD‐derived structural coordinates of the respective complexes. These parameters provide insights into the relaxation dynamics of SMMs. The Orbach relaxation process is assumed to occur via the excited state, where there is a substantial transverse magnetization present, meaning that the g_z_ vector starts to become perpendicular to the principal magnetic axis and the g_x_, g_y_ values increase significantly relative to the g_z_ values. This observation is consistent with both the calculated and observed *U*_eff_ values for all the complexes in Table 2, which are in close agreement. However, the observed blocking temperatures of these SMMs tell a different story. This is because the current state of computational chemistry of Ln SMMs is limited to only accurately estimating the Orbach process, and the faster Raman process is also known to be important for SMMs. Hence, we often do not observe the expected higher *T*_B_ values from Dy alkoxide and aryloxide SMMs, despite these examples having very high *U*_eff_ values.

Although Dy alkoxide and aryloxide SMMs with axial CF have provided some of the highest magnetic reversal energy barriers, to date their blocking temperatures have been far short of the leading examples of axial bis‐Cp^R^ (Cp^R^=substituted cyclopentadienyl, C_5_R_5_) Ln SMMs and their *bis*‐phospholyl counterparts.[^38^, ^104^, ^105^] Although most complexes in Table 2 show slow relaxation behaviour (i.e., temperature‐frequency dependence and a maximum in the out of phase susceptibility) up to appreciably high temperatures (>50 K), almost all of them have either significant QTM or a wide range of Raman relaxation processes in operation. The high energy barrier comes from the high‐temperature region in the fitting of the relaxation plots; this is where the Orbach process dominates. The reason behind selected highly axial bis‐Cp and *bis*‐phospholyl Ln SMMs outperforming Ln alkoxide SMMs in terms of their blocking temperature,[^13^, ^38^] is the hindrance of faster processes at low temperatures. Among comparable Dy aryloxide SMMs, the relaxation does not follow the thermal process, and the spin does not reach the higher excited states; instead, it tends to tunnel through the barrier. According to a recent inelastic neutron scattering study on [Dy(Cp^ttt^)_2_][B(C_6_F_5_)_4_] (Cp^ttt^=C_5_H_2_(*t*Bu_3_)‐1,2,4), the vibrational modes responsible for faster relaxation processes couple with the low‐energy acoustic phonons.^[19]^ At low temperatures when the spin does not have enough energy to relax via thermal processes, it utilises low‐energy vibrations by coupling to the acoustic phonons to relax. The vibrations present in the first coordination sphere of the compounds arise from the displacement of the Dy centres themselves. The rigid Cp^ttt^ ligands hold the Dy centre and dampen these vibrations, and consequently, the spins start to follow the thermal relaxation process, leading to higher blocking temperatures.

### 6.2 Enhancing molecular rigidity by ligand functionalisation

**Figure 17:**
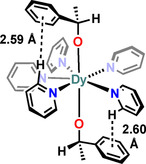
Depiction of [Dy(OR)_2_(py)_5_][BPh_4_] {OR=(*S*)‐(−)‐1‐phenylethanoxide} (**7.7**), showing the intramolecular C−H⋅⋅⋅π interactions.^[16]^

In high‐performing Ln alkoxide and aryloxide SMMs, low CN, featuring strong axial and weak equatorial donors, are common. A recent report by Yu et al. demonstrates a ligand functionalisation approach to enhancing magnetic hysteresis.^[16]^ The authors modified [Dy(O*t*Bu)_2_(py)_5_][BPh_4_] (**7.1**) by replacing the O*t*Bu ligand with a larger phenyl‐substituted ethoxide to yield [Dy(OR)_2_(py)_5_][BPh_4_] {OR=(*S*)‐(−)‐1‐phenylethanoxide} (**7.7**). The overall structure of **7.7** is similar to **7.1**, though the presence of axial phenyl groups leads to intramolecular C−H⋅⋅⋅π interactions with the pyridine co‐ligands, greatly enhancing the overall rigidity of the molecule (Figure 17). Moreover, there are also intermolecular C−H⋅⋅⋅π and π⋅⋅⋅π interactions within the crystal structure of **7.7**, which should have a direct influence on the packing and rigidity of the crystal lattice, altering the phonon bath and consequently the energy of lattice vibrations. As expected, the linear coordination environment of **7.7** yields a large *U*_eff_ (1625(28) K), which is lower than in **7.1** (1815 K). Interestingly, **7.7** shows magnetic hysteresis up to 22 K ($T_{B}^{Hyst}$ =4 K for **7.1** K), and has a higher $\tau_{switch}$ value of 0.22 s (0.060 s for **7.1**). Strangely, the experimental data shows that **7.7** relaxes faster than **7.1** in the Orbach region but is slower in the Raman region. Ab initio calculations reveal that the overall electronic structure of **7.7** is similar to **7.1**; the improvement in $T_{B}^{Hyst}$ is assigned to a difference in the phonon density of states. Additionally, relaxation data for a diamagnetically doped sample of **7.7** showed very similar Orbach and Raman behaviour to the pure sample, indicating that the variation in relaxation dynamics between **7.1** and **7.7** is not due to intermolecular dipolar interactions. Detailed spin dynamics calculations were performed to elucidate the contributions of molecular vibrations to magnetic relaxation.

To determine the transitions which are most important for fast magnetic relaxation in the Orbach region, Chilton et al. have developed a “knockout” procedure^[16]^ wherein they set all the possible transition probabilities out of the ground state (±15/2) to zero sequentially, while calculating the τ^−1^ versus *T* for each step. This “knockout” procedure revealed that the most important initial step of magnetic relaxation in **7.1** is the GS(‐15/2) to ES_2_(‐11/2) transition (Δ*E*=≈1300 K), which is coupled to the C−H deformation of the equatorial py co‐ligands (modes 95 and 96). However, the equivalent modes of **7.7** are not on resonance with the GS(‐15/2) to ES_2_(‐11/2) transition, and instead initial relaxation proceeds via the higher energy fifth excited state (Δ*E*=≈1400–1500 K) by coupling to modes involving the axial aryl groups. Therefore, the authors posited that, while the increased number of vibrational modes introduced by ligand functionalisation effectively boosted Orbach relaxation in **7.7**, it is the rigidity of the molecule as a whole which diminishes the effects of quantum tunnelling at zero‐field, improving the hysteresis temperature of **7.7** versus that of **7.1**.

## 7 Other Ln Alkoxide and Aryloxide SMMs

### 7.1 Mononuclear Er^III^ SMMs

Amongst Ln Kramers ions, apart from Dy^III^ only Er^III^ has a *J=*15/2 ground state (^4^I_15/2_). The best‐performing mononuclear Er^III^ SMMs in the literature to date is the Er^III^ COT family of SMMs, which have been reviewed elsewhere.[^106^, ^107^, ^108^, ^109^, ^110^] [Er(COT)(Cp*)] (COT=cyclooctatetraenyl, Cp*=pentamethylcyclopentadienyl) was the first mononuclear Er^III^ SMM, showing *U*_eff_≈323 K and $T_{B}^{Hyst}$ of 5 K as reported by Gao and co‐workers in 2011.^[107]^ More recently, Meng et al. reported [Er(COT)(C_5_H_5_BMe)] with the highest *U*_eff_ (422 K) among all the mononuclear Er^III^ SMMs.^[106]^

**Figure 18:**
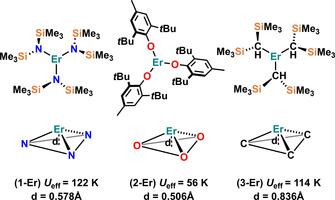
Molecular structures (1st row) and coordination geometries of the first coordination sphere (2nd row) in **1‐Er**, **2‐Er** and **3‐Er**; d is the distance of Er from the trigonal plane defined by the three donor atoms.^[111]^

The charge density distribution of the magnetic states of Er^III^ ions shows that, unlike Dy^III^, the strongest magnetic state (*m*_J_=15/2) of the Er^III^ ion forms a prolate spheroid.^[42]^ This suggests that highly anionic donors in a trigonal planar geometry about the Er^III^ centre can stabilise the ^4^I_15/2_ state as the magnetic ground state.[^6^, ^35^] A perfect *C*_3*h*_ symmetry would eliminate all the transverse CF terms except the sixth‐order transverse anisotropy *q*=±6, thereby restricting QTM.^[35]^ Bulky aryloxide ligands have demonstrated their effectiveness for stabilising three‐coordinate Ln complexes.[^111^, ^112^] However, in the literature, there are only three CN3 mononuclear Er^III^ SMMs reported to date[^111^, ^113^] (Figure 18). [Er{N(SiMe_3_)_2_}_3_] (**1‐Er**) was the first mononuclear three‐coordinate Er^III^ SMM to be reported;^[113]^ although this complex was first synthesised by Bradley in 1972^[114]^ its magnetic properties were only explored in 2014 by Zhang et al.^[113]^ The other two Er^III^L_3_ SMMs, [Er(OC_6_H_2_ *t*Bu_2_‐2,6‐Me‐4)_3_] (**2‐Er**) and [Er{CH(SiMe_3_)_2_}_3_] (**3‐Er**) were recently reported by Yamashita and co‐workers.^[111]^ Complex **2‐Er** is the only three coordinate Er^III^ aryloxide SMM; it shows a *U*_eff_ of 56 K, which is the lowest amongst mononuclear Er^III^L_3_ SMMs.^[111]^ This is attributed to the closer localisation of the electron density (from the donor lone pair) towards the Er^III^ centre in **3‐Er** and **1‐Er** compared to **2‐Er**.^[111]^

Despite being low coordinate, the mononuclear Er^III^L_3_ SMMs are outperformed by mononuclear Er^III^ COT SMMs. This is because the Er^III^L_3_ complexes **1**–**3‐Er** exhibit trigonal pyramidal geometries (Figure 18, 2nd row) which results in a non‐zero *q*=±3 CF term.[^111^, ^115^, ^116^, ^117^] The Er centres are located 0.578, 0.506 and 0.836 Å away from the three ligand donor atom planes in **1‐Er**, **2‐Er** and **3‐Er**, respectively. Recently, Lu et al. reported a theoretical investigation explaining the inability of mononuclear Er^III^ SMMs to generate huge energy barriers^[118]^ by studying **1**–**3‐Er**. The authors found that the calculated g_(*x*,*y*)_ values in the GS‐KDs (ground state Kramer's doublet) for complexes **1**–**3‐Er** are all close to zero, while those in ES_1_‐KDs are relatively large. The transverse magnetic moments in the GS‐KDs for **1**–**3‐Er** are of the order 10^−4^ *μ*_B_, respectively, which indicates the QTMs in their GS‐KDs could be suppressed at low temperature. However, transverse magnetic moments larger than 0.1 *μ*_B_ are large enough to cause QTM in the ES_1_‐KDs.[^118^, ^119^, ^120^] CF parameters in **1**–**3‐Er** indicated that only the second order axial parameters $B_{k=2}^{q=0}$ considerably exceed the nonaxial CF parameters $B_{k=2}^{q\neq 0}$ , which shows **1**–**3‐Er** have moderately axial symmetry. The significantly larger non‐zero g_(*x*,*y*)_ values of ES_1_‐KDs in **1**–**3‐Er** lead to transverse magnetic moments higher than 0.1 *μ*_B_, leading to enhanced QTM and restricting spin‐phonon transitions from GS to ES_1_ only.^[118]^ Using the computational model, the authors showed that even when the geometries of Er^III^L_3_ complexes are perfectly trigonal planar, the highest energy barrier in Er^III^L_3_ SMMs would be around 305 K. The Er‐L distance also does not affect the energy barriers in Er^III^L_3_ SMMs; decreasing the Er‐L distance did not increase the calculated *U*_eff_ due to the increasing probability of *m*_J_ state mixing among the GS and ESs.^[118]^ Hence, excluding any anharmonic spin‐phonon coupling and other second‐order two‐phonon processes, the realistic energy barriers in mononuclear Er^III^ SMMs would effectively be the GS‐ES_1_ gap.^[118]^ Four and five‐coordinate Er^III^ SMMs are known in the literature, but none of these contains alkoxides or aryloxides.[^116^, ^117^, ^121^] Among higher‐coordinate (CN>5) mononuclear Er^III^ alkoxide and aryloxide SMMs, none show improved SMM behaviour over the Er^III^ COT family or Dy^III^ derivatives of higher‐coordinate alkoxide and aryloxide SMMs.[^78^, ^79^, ^81^, ^85^, ^89^, ^122^, ^123^, ^124^, ^125^]

### 7.2 Mononuclear Ln SMMs of non‐Kramers Tb^III^ and Ho^III^ ions

Tb^III^ and Ho^III^ possess high magnetic spin ground states but are non‐Kramers ions; hence they are inefficient SMMs in the absence of an external magnetic field.^[28]^ Nevertheless, a small external magnetic field can initiate slow magnetic relaxation; hence the majority of Tb and Ho SMMs are field‐induced SMMs.[^126^, ^127^, ^128^] Besides the external field, an ideal molecular geometry causing appropriate local symmetry around the metal centre can also cause slow magnetic relaxation in mononuclear Tb and Ho compounds. The first mononuclear Ln SMM **TbPc2** showed relatively high energy barrier as it has a double‐decker structure with a square‐antiprismatic coordination geometry.^[5]^ Complex **TbPc2** and its various derivatives have greatly contributed to the understanding of the electronic structure and magnetostructural correlation in Ln SMMs and have been the most widely studied Ln SMMs to date.[^126^, ^127^, ^128^, ^129^, ^130^] There has been no Tb alkoxide or aryloxide SMM with CN <6 in the literature to date. Tb SMMs with higher CNs with mainly multi‐dentate chelating aryloxide ligands have been investigated.[^85^, ^95^, ^131^, ^132^, ^133^] However, they do not show a remarkable improvement over their Dy and Pc counterparts.[^130^, ^133^]

Ho^III^ SMMs remain relatively unexplored despite having the highest possible magnetic spin ground state (^5^I_8_: *m*_J_=±8) amongst all the Ln^III^ ions. The majority of Ho SMMs are multinuclear/3d‐4f compounds, and only a handful of mononuclear Ho^III^ SMMs are currently known.[^10^, ^134^, ^135^, ^136^, ^137^, ^138^, ^139^, ^140^] Zheng and co‐workers very recently reported a family of Ho^III^ alkoxide and aryloxide PB SMMs; [Ho(L)_2_(py)_5_][BPh_4_] {L=OSiMe_3_ (**7.1‐Ho**); OCH(Me)C_6_H_5_ (**7.2‐Ho**); OC_6_H_3_Me_2_‐2,6 (**7.3‐Ho**). Complex **7.1‐Ho** shows the highest *U*_eff_ value of 715(6) K amongst Ho SMMs to date; the *U*_eff_ values for **7.2‐Ho** and **7.3‐Ho** are 499(3) and 397(12) K, respectively.^[140]^ The molecular structures of **7.1–7.3‐Ho** are similar to [Dy(O*t*Bu)_2_(py)_5_][BPh_4_];^[13]^ the Ho‐O distances in **7.1–7.3‐Ho** range from 2.139(3)‐2.141(4) Å and the O‐Ho‐O angles from 174.51(14)‐176.12(7)°. AC magnetic susceptibility data revealed slow relaxation in zero applied field up to 43 K (**7.1‐Ho**), 34 K (**7.2‐Ho**) and 26 K (**7.3‐Ho**) at 10 KHz frequency. CASSCF‐SO calculations on **7.1‐Ho** showed a pure *m*_J_=±8 GS‐KD with a g_z_ value of 19.87, indicating highly axial ligand field and magnetic anisotropy. The GS‐ES_1_ gap for **7.1‐Ho** was calculated to be ≈500 K with a pure ES_1_‐KD=±7 showing minimal tunnelling probability. LoProp^[141]^ charge analysis indicated very similar charges on the equatorial donor atoms, so a similar CF operates in **7.1–7.3‐Ho**; however, the axial donor strength increased from **7.1‐Ho** to **7.2‐Ho** to **7.3‐Ho**, which justifies the same trend in the *U*_eff_ values. The remarkably high *U*_eff_ in **7.1‐Ho** was attributed to the pure state transition of *m*_J_=±8→*m*_J_=±7→*m*_J_=±6; hence the faster transitions start at KD_3_.^[140]^

### 7.3 Mononuclear Yb^III^ SMMs

Yb^III^ is a prolate‐type Ln^III^ ion, with a highest possible *m*_J_ level of 7/2 (^2^F_7/2_). Despite being a Kramers ion, there are only a few Yb^III^ SMMs in the literature compared to Dy^III^, Er^III^ and even non‐Kramers Tb^III^ ions.[^27^, ^142^] Almost all the Yb^III^ SMMs require an external magnetic field to show slow magnetic relaxation.[^85^, ^103^, ^108^, ^133^, ^142^, ^143^] Owing to the possibility of ferromagnetic exchange interactions, the vast majority of Yb^III^ SMMs are either homometallic dimers or heterometallic 3d‐4f mixed multinuclear complexes, which have been discussed in detail elsewhere.^[142]^ Mononuclear Yb^III^ SMMs most commonly show CN8; the majority of them contain large multi‐dentate ligands.[^85^, ^103^, ^133^, ^142^] Sujita et al. reported the first field‐induced mononuclear Yb^III^ SMM, [NEt_4_]_3_[Yb(dipic)_3_] (dipic=pyridine‐2,6‐dicarboxylate) (**1‐Yb**) in 2006.^[144]^ The coordination sphere of **1‐Yb** contains nine donor atoms in a N_3_O_6_ configuration, with a reported *U*_eff_ of 187 K at 1000 Oe applied field. In 2012, Tong et al. reported a six‐coordinate mononuclear Yb^III^ aryloxide SMM, [Yb(H_3_L1^tren^)_2_]Cl_3_ (**2‐Yb**) {H_3_ L1^tren^=tris(((2‐hydroxy‐3‐methoxybenzyl)amino)ethyl)‐amine}.^[145]^ The Yb^III^ ion in **2‐Yb** adopts a distorted octahedral geometry, which is amongst the few low coordinate mononuclear Yb^III^ SMMs; this complex was reported to have a *U*_eff_ of 6–7 K under 400 Oe external field.^[145]^ It is to be noted here that the *U*_eff_ values do not properly represent the relaxation dynamics of such systems and now it appears that relaxation is most likely through a combination of Raman and QTM process rather than via an Orbach relaxation.

Yb^III^ SMMs often contain multifunctional ligands and metal centres that are capable of showing luminescence behaviour, which is beneficial for studying their ground and excited magnetic state energy levels via spectroscopy.[^78^, ^103^, ^142^, ^143^] In 2009, Huang et al. utilised the functionalised rhodamine‐incorporated quinolinolate ligand RhQ (rhodamine‐6G‐2‐(hydrozinomethyl) quinoline‐8‐ol) to design and synthesise a near‐infrared (NIR) luminescent mononuclear Yb^III^ SMM [Yb(RhQ)_2_][NO_3_] (**3‐Yb**).^[146]^ The ring‐opened rhodamine unit provides the energy of excitation in the visible range (500 nm).^[146]^ Similarly, in 2015, Huang et al. utilised the same ligand system along with a strong co‐ligand (tta^−^) to synthesise [Yb(RhQ)(tta)] (**4‐Yb**), which is isostructural with **8.36**.^[103]^ Both **3‐Yb** and **4‐Yb** have the CN8 Yb^III^ centres, showing *U*_eff_ values of 5–6 and 164 K at 1000 Oe, respectively.[^103^, ^146^]

The large multi‐dentate trianionic trensal {H_3_trensal=2,2′,2“‐tris(salicylideneimino)triethylamine)} ligand has been widely utilised for encapsulating Ln^III^ ions in the synthesis and various magnetic studies of mononuclear Ln^III^ SMMs, including Yb.[^28^, ^147^, ^148^, ^149^] [Yb(trensal)] has been proposed as an electronic qubit for developing quantum information processing (QIP) devices.[^150^, ^151^] The most recent Yb^III^ SMMs containing functionalised trensal ligands were reported by Buch et al. in 2020.[^133^, ^152^] In one study, Buch et al. functionalised the trensal ligands with appropriate groups to enable surface deposition and synthesised [Yb(L)] (L=tris(((3‐formyl‐5‐methylsalicylidene)amino)ethyl)amine; **5‐Yb**).^[133]^ The hepta‐coordinated Yb^III^ centre in **5‐Yb** has a similar local symmetry to other [Ln(trensal)] complexes.[^28^, ^147^, ^148^, ^149^] The benzylamine‐functionalised trensal ligand in **5‐Yb** contains anchoring groups that can bind to surfaces, enabling surface deposition of Ln SMMs to study QIP.^[133]^ Complex **5‐Yb** did not show SMM behaviour under zero applied field; upon applying a 2000 Oe field, it showed slow relaxation of magnetisation. However, due to instrument limitations, the authors could not quantify the SMM behaviour as the magnetic relaxation is governed by a direct process at low temperatures and a Raman process at higher temperatures, like other SMMs related to [Yb(trensal)].[^28^, ^147^, ^148^, ^149^]

Although, Yb^III^ complexes are often weak field‐induced SMMs, they have been proposed as qubits for quantum computers, and being applied to nano‐device development and understanding molecular spintronics.[^3^, ^150^, ^151^] This is due to the unique properties of mononuclear Yb^III^ SMMs such as photoluminescence, large splitting between the ground and excited KDs, and controllable slow relaxation of magnetisation via external field, which promotes longer coherence time by restricting any hindrance from spin‐lattice relaxation.[^85^, ^142^, ^143^, ^151^] Moreover, the ground KD of the *J=*7/2 ground term of Yb^III^ (^2^F_7/2_) can be approximated as an effective spin −1/2 system in a wide temperature interval, making it suitable to study coherent manipulation of the electron‐spin by EPR protocols.^[151]^ From a pure SMM perspective, the properties of the best performing Yb^III^ SMMs are far behind those of leading Dy^III^ SMMs, and more studies and investigations are needed to understand their relaxation dynamics to promote zero‐field slow relaxation of magnetisation at accessible temperatures. For further understanding of SMMs of uncommon Ln^III^ ions (Yb^III^, Ce^III^, Nd^III^, Ho^III^ and Tm^III^), readers are directed to the 2017 review on this subject by Pointillart et al.^[142]^

## 8 Conclusion

An ideal candidate for a high‐performance Dy alkoxide or aryloxide SMM would be a linear two‐coordinate mononuclear complex. However, as the oxygen atoms of these ligands can only bear one R or Ar group, steric protection of the equatorial coordination sites or control of the bend angle of a theoretical [RO‐Dy‐OR]^+^ cation is more challenging for alkoxides and aryloxides than for monodentate anionic ligands with additional R‐groups (e.g., alkyl, CR_3_; amide, NR_2_), or aromatic anionic ligands (e.g., Cp^R^, C_5_R_5_). These limitations have motivated studies to explore alternative approaches, whereby weakly donating ligands are allowed to occupy the equatorial positions, and in an ideal situation imposing a O‐Dy‐O angle of 180°. Consequently, many recently reported mononuclear Dy alkoxide and aryloxide SMMs with higher coordination numbers have shown remarkable energy barriers to magnetic reversal and show capacity for this parameter to be enhanced even further. These complexes generate highly uniaxial ligand fields whilst providing appreciable control over the molecular geometry. Reducing the equatorial donors to softer and more charge‐diffuse alternatives should enhance uniaxial ligand fields and *U*_eff_ values further by reducing the transverse CF and consequently QTM.

Even though the blocking temperatures of highly axial mononuclear Dy alkoxide and aryloxide SMMs are not yet in the same league as leading examples of axial *bis*‐Cp^R^ Ln systems and their *bis*‐phospholyl counterparts, they have provided sufficient geometrical control to study and explore the relaxation dynamics of SMMs.^[14]^ Understanding the relaxation dynamics of SMMs is crucial to allow us to eventually design and develop SMMs that can show magnetic blocking at higher temperatures. Alongside the geometrical control, most of the mononuclear Dy alkoxide SMMs discussed herein show all three major relaxation processes in their relaxation profiles, which makes them interesting systems for further study. Controlled intra‐ and inter‐molecular interactions such as C−H⋅⋅⋅π interactions^[16]^ can provide extra rigidity to these structures. Fine‐tuned interactions in the crystal can dampen the vibronic couplings responsible for faster relaxation times. Under controlled and optimised geometries, Dy alkoxide SMMs where the ligand donor atoms are substituted with heavier alternatives (e.g., siloxides)^[14]^ could provide further insights into the effects of CF, the nature of the Dy−O bond, and the covalent factors influencing the magnetic properties of high‐performing SMMs.^[153]^

## Note added in proof

Following the submission of this article, a series of eight CN5 mononuclear Dy^III^ alkoxide and aryloxide SMMs were reported; the Dy^III^ centres in each of these complexes exhibit square‐based pyramidal geometries and variation of the apical ligand was found to affect the SMM properties,^[154]^ similar to that discussed for reference [15].

## Appendix

List of abbreviations:

Dipp: 2,6‐diisopropylphenyl

acac: acetylacetonate

Dppd: dibenzoylmethane

O*t*Bu: *tert‐*butoxide

OPh: phenoxide

ODipp: 2,6‐diisopropyl phenoxide

ODPP: 2,6‐diphenyl phenoxide

ODMP: 2,6‐dimethyl phenoxide

ODBP: 2,6‐di‐*tert*‐butyl phenoxide

ONap: naphthoxide

OMes: 2,4,6‐trimethyl phenoxide

OMes*: 2,4,6‐tri‐*tert‐*butyl phenoxide (two relaxation processes)

ODPhMP: 2,4‐bis‐diphenyl‐4‐methyl phenoxide

O^*i*Pr^ter‐Ph: 2,6*‐*Dipp_2_ phenoxide

O^*i*Pr^ter‐Ph′′^:^ 6‐Dipp‐2‐(2′‐*i*Pr‐6′‐CHMe(CH_2_)‐C_6_H_3_) phenoxide

ONPh: 4‐nitro phenoxide

OCl_2_NPh: 2,6‐dichloro‐4‐nitro phenoxide

3‐NO_2_‐salenH_2_: *N*,*N*′‐bis(3‐nitro‐salicylaldehyde)ethylenediamine

H_3_OR‐tripamine: tris((2‐hydroxy‐5‐*n*‐butylbenzoate)aminoethyl)amine

ODBquPh: 2,4‐di‐*tert*‐butyl‐6‐((quinolin‐8‐ylimino)methyl)phenolate

ONpyPh: 2‐(6‐(hydroxymethyl)pyridin‐2‐yl)‐methyleneamino)phenol

H_2_bpen: *N*,*N*′‐bis(2‐methylenepyridinyl)ethylenediamine

H_2_bbpen: *N*,*N*′‐bis(2‐hydroxybenzyl)‐*N*,*N*′‐bis(2‐methylpyridyl)ethylenediamine)

bbpen^MeCHO^: *N*,*N*′‐bis(2‐hydroxy‐5‐methyl‐3‐formylbenzyl)‐*N*,*N*′‐bis(pyridin‐2‐ylmethyl)ethylenediamine

(*R*,*R*)/(*S*,*S*)‐3‐NO_2_‐salenH_2_: *N*,*N*′‐(1,2‐cyclohexanediylethylene)bis(3‐nitrosalicylideneiminato)

H_4_N_4_TzcA: *N*,*N*′,*N*′′,*N*′′′‐tetra(3,5‐dimethyl‐2‐hydroxybenzyl)‐1,4,7,10tetraazacyclododecane

HOAr^8^⋅^21^: 2‐(5‐bromo‐3‐((2‐(dimethylamino)ethyl)methylamino)methyl)‐2‐hydroxyphenyl)‐4,4,5,5‐tetramethyl‐1*H*‐3l4,4l5,5l5‐imidazole‐1,3‐bis(olate)

HOAr^8^⋅^22^: (*E*)‐2‐(3‐((bis(pyridin‐2‐ylmethyl)amino)methyl)‐2‐hydroxy‐5‐nitrostyryl)‐1,3,3‐trimethyl‐1*H*‐3l5‐indol‐1‐ium

N_6_O_3_Ar_*R*_: (4^1R^,4^2S^,10^1R^,10^2S^,16^1R^,16^2S^)‐15,75,135‐trimethyl‐3,5,9,11,15,17‐hexaaza‐1,7,13(1,3)‐tribenzena‐4,10,16(1,2)‐tricyclohexanacyclooctadecaphane‐12,72,132‐triol

N_6_O_3_Ar_*S*_: (4^1S^,4^2R^,10^1S^,10^2R^,16^1S^,16^2R^)‐15,75,135‐trimethyl‐3,5,9,11,15,17‐hexaaza‐1,7,13(1,3)‐tribenzena‐4,10,16(1,2)‐tricyclohexanacyclooctadecaphane‐12,72,132‐triol

tta: 2‐thenoyltrifluoroacetone

tfa: 1,1,1‐trifluoroacetylacetone

tmpd: 4,4,4‐trifluoro‐1‐(4‐methoxyphenyl)‐1,3‐butanedione

DiAmino‐tz: 2,4‐diamino‐6‐pyridyl‐1,3,5‐triazine

dsp: disalicylidene‐1,2‐phenylenediamine

tpe‐COOH: 4′‐(1,2,2‐triphenylvinyl)benzoic acid

H_2_Pc: metal‐free phthalocyanine

Q: quinolinolate

MQ: 2‐methyl quinolinolate

Cl_2_MQ: 5,7‐dichloro‐2‐methyl quinolinolate

Br_2_MQ: 5,7‐dibromo‐2‐methyl quinolinolate

Me_2_Q: 5,7‐dimethyl quinolinolate

Cl_2_Q: 5,7‐dichloro quinolinolate

LQ^CO^: 8‐hydroxyquinoline‐2‐carboxaldehyde‐(aminourea)hydrochloride

HnmQ: *N*‐(2‐(8‐hydroxylquinolinyl)methane(2‐(4‐imidazolyl)ethanamine))

ClNPhQ: 2‐[(4‐chlorophenyl)imino]methyl]‐8‐hydroxyquinoline)

HpyQ: *N*‐(methylene‐8‐hydroxyquinoline)‐pyridylhydrazone)

RhQ: rhodamine‐6G‐2‐(hydrozinomethyl) quinolin‐8‐oate

ODBpyPh: 2,4‐di‐*tert*‐butyl‐6‐((pyridin‐2‐ylmethyl)imino)methyl)phenolate

L^CO^H: *N*‐[(2‐MeO)C_6_H_5_]}N=C(Me)CH=C(Me)N(H){N′‐[(2‐MeO)C_6_H_5_]

THF: tetrahydrofuran

py: pyridine

Phpy: 4‐phenylpyridine

DMpy: 3,5‐dimethylpyridine

Plpy: 4‐pyrrolidin‐1‐ylpyridine

Pdpy: 1‐4‐piperidin‐1‐ylpyridine

HN′′: HN(SiMe_3_)_2_

## Conflict of interest

The authors declare no conflict of interest.

## Biographical Information

*Vijay S. Parmar was awarded his BS‐MS dual degree in 2016 from The Indian Institute of Science Education and Research (IISER), Bhopal, India. His PhD, jointly supervised by Dr David P. Mills and Prof. Richard E. P. Winpenny, focuses on the development and characterisation of low‐coordinate lanthanide single‐molecule magnets and investigating their magnetic relaxation dynamics*.

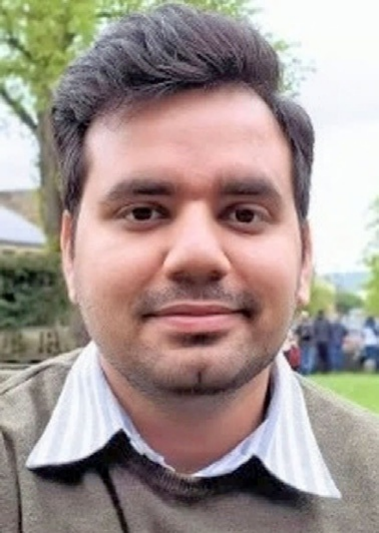

## Biographical Information

*David P. Mills is a Reader at the Department of Chemistry in the University of Manchester. His research interests are currently focused on the synthesis of lanthanide and actinide complexes with unusual geometries, oxidation states, and bonding arrangements, which have been applied to the design of high‐temperature Ln SMMs*.

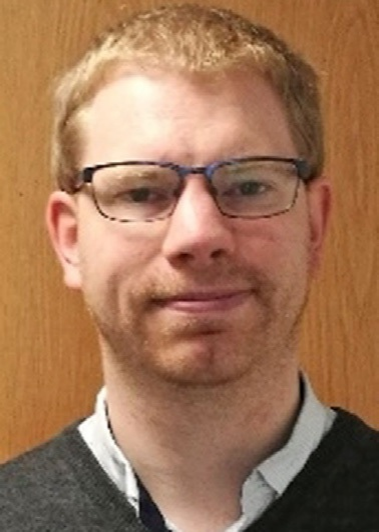

## Biographical Information

*Richard E. P. Winpenny is a Professor of Inorganic Chemistry at the Department of Chemistry in the University of Manchester. His research focuses on synthesis and study of SMMs, large supramolecular rings, qubits and nanomaterials. He has won many distinguished awards (Royal Society Wolfson Merit Award 2009; Royal Society of Chemistry Tilden Medal 2011 and Ludwig Mond Prize in 2016) in recognition of his research in molecular magnetism. He is also on the Board of Directors of the European Institute for Molecular Magnetism*.